# Polyphenol‐Inspired Materials for Agricultural Applications

**DOI:** 10.1002/adma.74575

**Published:** 2026-08-19

**Authors:** Haofu Liu, Omid Mazaheri, Zhixing Lin, Tianzheng Wang, Jingqu Chen, Yuang Gu, Xia Liang, Deli Chen, Frank Caruso

**Affiliations:** ^1^ School of Agriculture Food and Ecosystem Sciences Faculty of Science The University of Melbourne Parkville Victoria Australia; ^2^ Department of Chemical Engineering The University of Melbourne Parkville Victoria Australia; ^3^ ARC Research Hub for Smart Fertilisers The University of Melbourne Parkville Victoria Australia; ^4^ Department of Chemical and Petroleum Engineering Research and Innovation Center for Graphene and 2D Materials Food Security and Technology Center Khalifa University Abu Dhabi UAE

**Keywords:** advanced materials, agricultural applications, crop production, plant–soil interactions, sustainable agriculture

## Abstract

Securing global food production while reducing environmental burdens demands materials that combine high nutrient use efficiency with sustainability. Polyphenols, a class of natural plant‐derived molecules, provide redox activity, multidentate interactions, and strong interfacial adhesion, making them versatile building blocks for bio‐derived agricultural systems. This review summarizes recent advances in the molecular design and multifunctional applications of polyphenol‐inspired materials across diverse agriculture sectors, including soil remediation, seed coating, nutrient delivery, crop protection, sensing, nitrification inhibition, and food preservation. It focuses on interfacial assembly, structure–property relationships, and environmental interactions of polyphenol‐enabled materials, which collectively govern their performance from laboratory tests to field conditions. Key challenges in current agricultural practice—including low precision and high labor dependence, environmental degradation and ecological imbalance, instability under extreme environmental conditions, and low economic efficiency and unsustainability—are also discussed. Finally, future directions centered on precision and smart agriculture, ecosystem protection, climate‐resilient plant interfaces, and circular bioeconomy are outlined. This review presents a comprehensive framework that connects molecular innovation to system‐level applications, offering a roadmap for future research and the deployment of polyphenols in agriculture.

## Graphical abstract

This review outlines the use of polyphenol‐inspired materials for sustainable agriculture, highlighting their molecular design, interfacial assembly, structure–property relationships, and prospects toward precision agriculture, climate resilience, ecosystem protection, and circular bioeconomy strategies. Their agricultural applications include soil remediation, seed coating, nutrient delivery, crop protection, sensing, nitification inhibition, and food preservation.

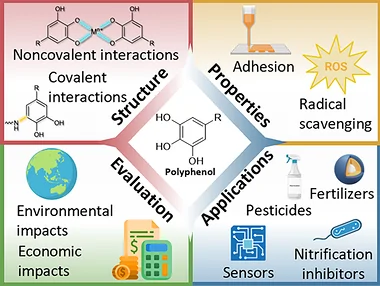

## 1 Introduction

Global agriculture faces unprecedented challenges driven by population growth, resource scarcity, and climate change, while remaining central to global food security and economic stability [1]. However, food production is increasingly constrained by environmental degradation and limited natural resources. Intensive land use, excessive agrochemical inputs, and declining water availability have disrupted nutrient cycling, degraded soil fertility, and increased greenhouse gas emissions [2]. These pressures collectively undermine the resilience of the ecosystem and threaten the long‐term sustainability of global food production. Although the use of synthetic agrochemicals (e.g., fertilizers and pesticides) has significantly increased crop yield, their use has also had unintended environmental consequences, including soil acidification, water eutrophication, and heavy metal accumulation [3, 4]. These environmental burdens conflict with sustainable agriculture principles, highlighting the urgent need for materials that sustain food productivity while protecting ecosystem health.

Traditional synthetic materials and chemical formulations have played a vital role in modern agriculture. For example, nano‐pesticides, controlled‐release fertilizers (CRFs), and smart delivery systems have improved nutrient use efficiency and crop productivity [5, 6]. However, these materials are largely derived from non‐renewable petroleum resources and exhibit poor biodegradability, leading to persistent residues and secondary pollution. Consequently, although petrochemical‐based inputs may improve short‐term yield, they compromise long‐term sustainability. To mitigate these impacts, bio‐derived materials have been explored as renewable alternatives [7, 8]. Their natural abundance, biocompatibility, and inherent degradability offer advantages over synthetic materials [9]. The development of bio‐derived materials (e.g., polysaccharides, proteins, and lipids) has led to advanced agricultural formulations and functional systems designed to improve resource use efficiency, reduce environmental impact, and enhance long‐term soil resilience [1, 6, 10]. However, their low mechanical stability, limited chemical tunability, and instability under complex environments (e.g., fluctuating pH, temperature, and humidity) restrict their application in agriculture [11].

Polyphenols are a distinctive class of naturally abundant molecules that have recently attracted increasing attention as multifunctional building blocks for the development of sustainable materials [12]. They are defined as organic compounds that contain multiple phenolic groups (i.e., aromatic rings bearing hydroxyl groups), regardless of the hydroxyl substitution pattern on the benzene ring (e.g., phenol, catechol, or galloyl moieties) [13, 14]. In this review, the term polyphenol refers to phenolic compounds containing at least one hydroxyl group on an aromatic ring [15]. These compounds, originating from diverse plant sources, exhibit rich redox activity and near‐universal binding affinity toward a wide range of guest molecules, including metal ions, polymers, and proteins [12]. Polyphenols can assemble into adaptive networks with reversible cross‐linking, strong adhesion, structural flexibility, and functional diversity through noncovalent interactions, redox chemistry, and dynamic covalent bonding with specific molecules (e.g., boric acid, aldehydes) [16]. Furthermore, the antioxidant and antimicrobial properties inherent in polyphenols help protect crops and nutrients from oxidative stress and microbial invasion, while their chelating ability to metal ions enables stabilization of nutrients and immobilization of toxic metal ions in soil [17]. The abundance, renewability, and environmental compatibility of polyphenols make them promising molecular scaffolds for developing sustainable agricultural materials capable of functioning under complex field conditions. However, their variations in molecular composition and functional groups can result in differences in reactivity, coordination behavior, and bonding interactions, leading to inconsistent material formation and performance. In addition, polyphenols can oxidize, presenting challenges for achieving consistent performance for use in agriculture (e.g., pesticides and nutrient‐controlled release).

To address these limitations, recent studies have focused on optimizing the chemical reactivity and structural organization of polyphenols through tailored modification and hybridization strategies [12]. Covalent functionalization with functional groups (e.g., silane, aldehyde, or acyl groups) enhances water resistance, mechanical robustness, and processing stability by introducing controlled cross‐linking sites [18]. Coordination assembly with multivalent metal ions (e.g., Cu^2+^, Fe^3+^, Ti^4+^, and W^6+^) provides stimuli‐responsive and dynamic networks that display self‐healing and antioxidative properties [12, 19, 20]. Hybridization with biodegradable components (e.g., small molecules, biomacromolecules, and polymers) has further improved flexibility and environmental compatibility, enabling composite systems that retain mechanical integrity [21, 22, 23]. Furthermore, encapsulation and self‐assembly approaches have been applied to stabilize reactive polyphenols and enable the sustained release of nutrients or bioactive molecules [24, 25, 26]. These advances collectively demonstrate that rational chemical and structural design, together with the assembly of guest molecules, can effectively overcome the limitations of polyphenols, enabling the development of durable and multifunctional materials for sustainable agriculture. Recent reviews have summarized the use of polyphenol‐based materials in agricultural applications, particularly in pesticides, fertilizers, and food preservation. However, a systematic understanding of how polyphenol molecular structures and assembly pathways govern material properties and agricultural performance remains limited [27, 28]. Specifically, the interrelationships between the chemistry of polyphenols, assembly strategies, and functional properties of engineered materials have not been established in the context of agricultural performance or broader environmental and economic assessments. Thus, a comprehensive review of recent advances in molecular design, modification, and assembly strategies of polyphenol‐enabled materials, along with their structure–property relationships and their performance in agriculture‐related applications, is needed to bridge these knowledge gaps and guide material design and optimization for diverse agricultural technologies.

This review aims to provide an overview of polyphenol‐enabled materials in sustainable agriculture, with a particular focus on the relationships between molecular design, material properties, and agricultural performance (Figure 1). First, the molecular design and assembly strategies of polyphenol‐enabled materials are outlined, followed by a discussion of their key material characteristics. Next, agricultural applications are highlighted, followed by a discussion on the environmental and economic implications of polyphenol‐enabled materials. Specifically, Section 2 introduces the sources, molecular structures, and core features of polyphenols and also provides a comprehensive discussion on their synthesis strategies, adhesion mechanisms, encapsulation capabilities, and tunable functionalities. Section 3 highlights recent advances in the development of polyphenol‐inspired material platforms for agricultural applications, including soil remediation, seed coating, the controlled release of pesticides and nutrients, sensing, nitrification inhibition, food preservation, and large‐scale implications. Section 4 summarizes the environmental and economic implications of polyphenol‐inspired materials. Section 5 concludes with a discussion on current challenges in agriculture and future prospects for polyphenol‐inspired materials in agricultural applications. The mechanistic insights and design principles derived from this review are envisioned to underpin the development of next‐generation polyphenol‐inspired materials for agricultural innovation.

**FIGURE 1:**
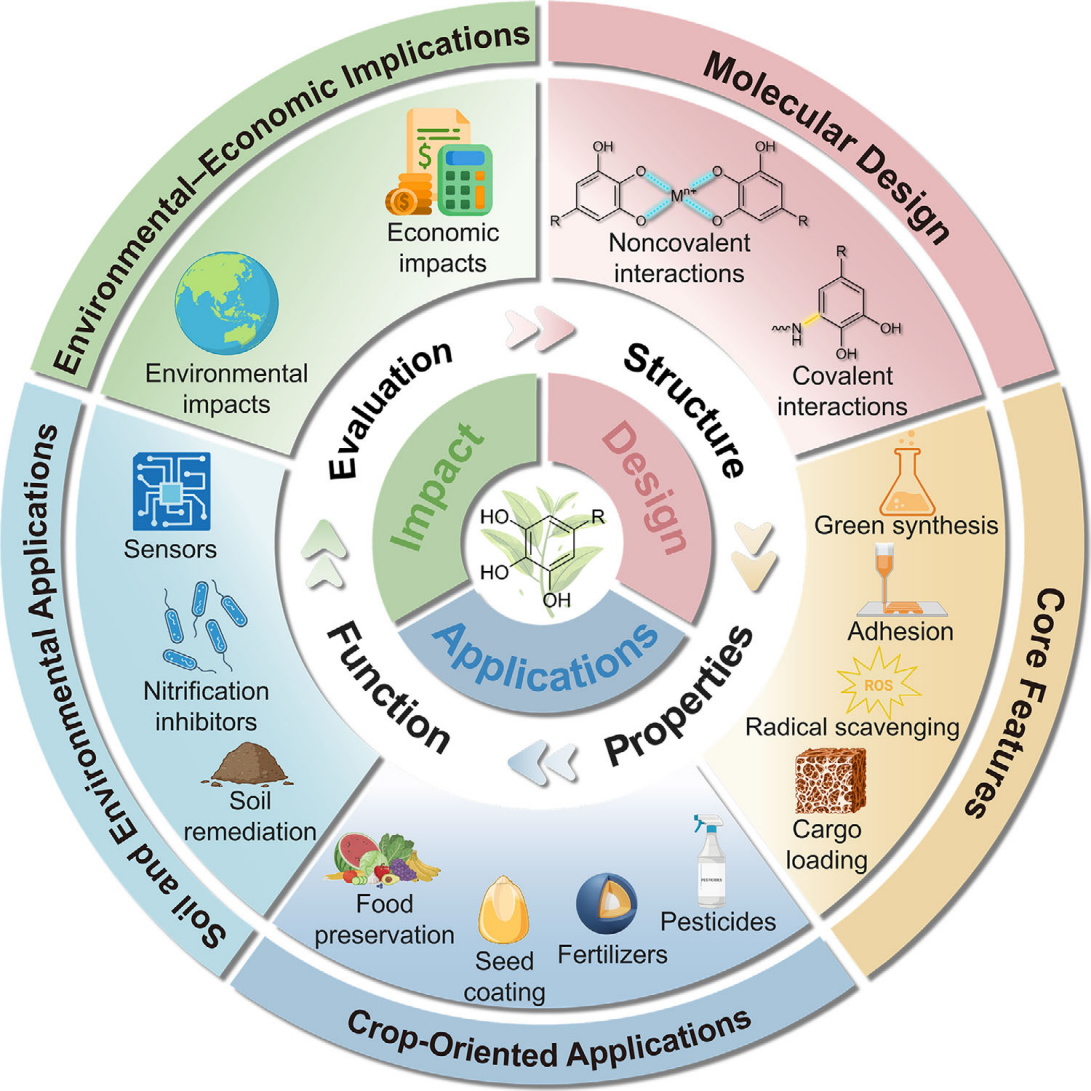
Schematic of the molecular structure design, core features, crop‐oriented applications, soil and environmental applications, and environmental–economic implications of polyphenol‐inspired materials. Created in BioRender.com. Liu, H. (2026) https://BioRender.com/udfivtg.

## 2 Polyphenols: Sources, Structures, and Features

Polyphenols are a broad family of plant‐derived secondary metabolites characterized by multiple hydroxyl groups with different substitution patterns on the aromatic ring (e.g., phenol, catechol, or galloyl moieties) and diverse molecular backbones. In this section, we introduce the main molecular sources and representative structures of polyphenols (Figure 2), followed by a discussion of their core features (i.e., strong adhesion, mild synthesis, versatile encapsulation, and tunable functionalities).

**FIGURE 2:**
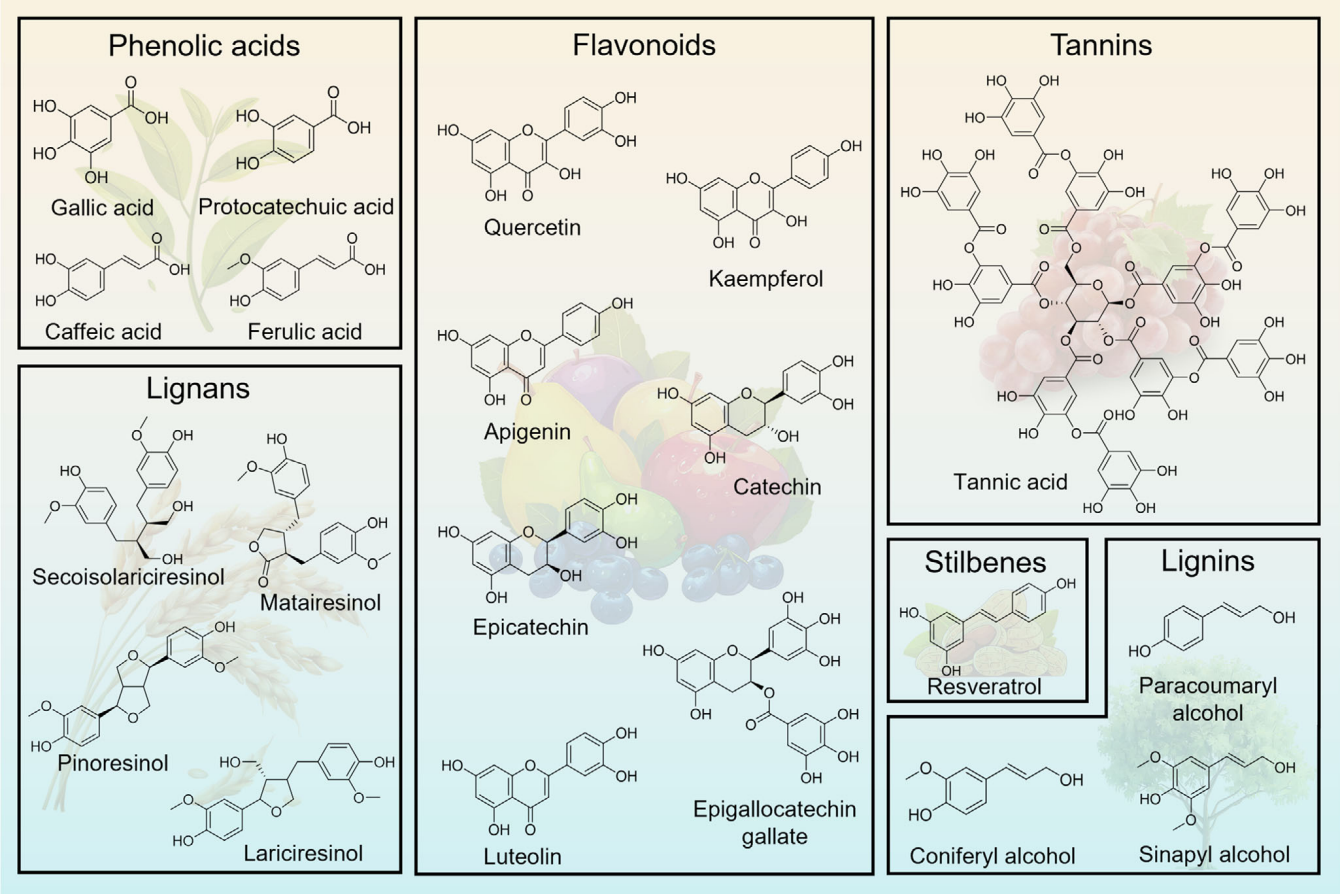
Chemical structures and primary sources of representative polyphenols: phenolic acids, flavonoids, tannins, lignans, stilbenes, and lignins.

### 2.1 Category of Polyphenols

#### 2.1.1 Phenolic Acids

Phenolic acids are a widely distributed subclass of plant polyphenols, structurally characterized by a phenolic ring with a carboxyl group. They are abundant in tea, berries, nuts, coffee, and vegetables. Based on the carbon backbone, phenolic acids are commonly classified into hydroxybenzoic acids and hydroxycinnamic acids [29]. Representative hydroxybenzoic acids include gallic and protocatechuic acids, whereas caffeic and ferulic acids are typical hydroxycinnamic acids. Phenolic acids frequently occur in bound forms, conjugated to sugars or linked to structural polymers such as lignin, which strongly influences their extractability, reactivity, and functionality [30]. The number and position of the hydroxyl groups in polyphenols govern their redox activity and metal coordination behavior, underpinning their roles in antioxidant protection, interfacial interactions, and coordination‐driven material assembly [31].

#### 2.1.2 Flavonoids

Flavonoids are a structurally diverse class of plant polyphenols, with over 4000 structures identified in nature. Based on differences in oxidation state and ring substitution, they are commonly classified into flavonols (e.g., quercetin), flavones (e.g., apigenin), flavanones (e.g., flavanone), and flavan‐3‐ols (e.g., epicatechin) [32]. These compounds are abundant in fruits, vegetables, tea, cocoa, and wine, where they contribute to color, flavor, and protection against oxidative stress [33, 34]. In plants, they often function as pigments and defense molecules. They are associated with broad health‐promoting effects, including antioxidant, anti‐inflammatory, and antimicrobial effects in humans [35, 36]. Flavonols, such as quercetin and kaempferol, provide multiple coordination sites that support redox‐active and mechanically adaptive assemblies [37]. Flavan‐3‐ols, exemplified by catechin and epicatechin, contain catechol motifs that underpin strong surface adhesion and dynamic metal–ligand interactions, while galloylated derivatives, such as epigallocatechin gallate (EGCG), further enhance chelation strength and network stability [38]. These features have been widely exploited in metal–phenolic assemblies, polymer cross‐linking, and surface coatings, where flavonoids contribute to tunable mechanical properties, antioxidant functionality, and environmental adaptability [39].

#### 2.1.3 Tannins

Tannins are an important subclass of polyphenols widely distributed in fruits, bark, seeds, and leaves, and are recognized for their strong antioxidant, antimicrobial, and protein‐binding properties [40]. According to their structural features, tannins are generally classified into hydrolysable tannins, condensed tannins, and phlorotannins, each exhibiting distinct chemical stability and interaction modes relevant to material construction [41]. Hydrolysable tannins consist of a polyol core esterified with gallic or ellagic acid units and can be readily cleaved under hydrolytic conditions [42]. Gallotannins contain multiple galloyl groups, whereas ellagitannins arise from oxidative coupling of galloyl units to form hexahydroxydiphenoyl motifs that can lactonize into ellagic acid [43, 44]. Their high phenolic group density enables strong hydrogen bonding, redox activity, and metal‐chelation capacity, which are central to dynamic cross‐linking and surface adhesion in polyphenol‐based materials. Condensed tannins, also termed proanthocyanidins, are dimeric, oligomeric, and even polymeric assemblies of flavan‐3‐ol units linked mainly through C─C bonds, conferring high resistance to hydrolysis and enhanced structural stability [45]. Phlorotannins are a distinct tannin family predominantly found in brown algae and are composed of polymerized phloroglucinol units linked via C─C or C─O─C bonds [46]. Their high hydroxyl content and flexible linkage patterns impart strong antioxidant and metal‐binding properties, making them attractive building blocks for functional materials [47].

#### 2.1.4 Lignans

Lignans are plant‐derived polyphenols formed by the dimerization of two phenylpropanoid units and typically contain multiple hydroxyl and methoxy groups [48]. Their metabolic conversion into enterolignans has been associated with antioxidant and anti‐inflammatory activities, contributing to various biological functions [49]. Secoisolariciresinol, mainly present as secoisolariciresinol diglucoside in flaxseed, represents one of the most abundant lignans [50]. Secoisolariciresinol diglucoside can be metabolized into enterodiol and enterolactone, which retain phenolic groups and antioxidant activity [50]. Matairesinol and pinoresinol, found in flaxseed, sesame, olive oil, and cereals, share similar dimeric architectures and redox characteristics [49, 51]. The rigid dimeric structure and multiple phenolic functionalities of lignans offer potential for redox‐mediated interactions and hydrogen bonding. Compared with tannins and flavonoids, lignans remain less explored as primary building blocks in engineered polyphenol‐enabled agricultural materials. This may be attributed to their relatively lower density of phenolic functional groups, which can limit the number of reactive sites available for material assembly and modification. However, the presence of methoxy groups in lignans provides opportunities for their further functionalization, facilitating their development into functional materials for agricultural applications.

#### 2.1.5 Stilbenes

Stilbenes are a relatively small subclass of polyphenols characterized by a 1,2‐diphenylethylene backbone, existing mainly in *cis*‐ and *trans*‐isomeric forms, with the *trans* configuration being thermodynamically more stable and biologically relevant [52]. They are synthesized through the shikimate pathway in plants and function primarily as phytoalexins, providing defense against pathogens and environmental stress [53]. Natural sources include grapes, peanuts, berries, and pine, with resveratrol being the most extensively studied representative [54]. Resveratrol and related stilbenes exhibit antioxidant activity derived from their conjugated aromatic structure and phenolic groups, enabling efficient radical scavenging and redox regulation [55]. Structural analogues, such as piceatannol, pterostilbene, and oxyresveratrol, share similar stilbene frameworks, with variations in hydroxylation and methoxylation affecting solubility and stability [56, 57]. The rigid aromatic backbone and redox‐active phenolic motifs of stilbenes provide potential for antioxidative protection and interfacial interactions. However, their relatively low aqueous solubility and limited multidentate binding capacity restrict their direct use as primary structural components in polyphenol‐based agricultural materials [52]. Moreover, stilbene derivatives are relatively expensive compared to other polyphenols, which limits their widespread use in agricultural studies, particularly because most agricultural applications require large‐scale and cost‐effective deployment.

#### 2.1.6 Lignins

Lignins are the most abundant aromatic biopolymers in nature after cellulose, accounting for approximately 15%–30% of plant biomass and constituting a major component of secondary cell walls [58]. They impart rigidity, hydrophobicity, and resistance to microbial degradation, thereby supporting mechanical strength and vascular transport in terrestrial plants [59]. Structurally, lignins are formed through the oxidative polymerization of three primary monolignols: *p*‐coumaryl, coniferyl, and sinapyl alcohols, which give rise to *p*‐hydroxyphenyl, guaiacyl, and syringyl units, respectively [60]. Variations in the relative abundance of lignin structural units across plant species and tissues strongly influence the chemical reactivity, condensation degree, solubility, and stability of lignins [61, 62, 63]. Higher aromatic conjugation in the backbones and phenolic groups of lignins confer intrinsic antioxidant activity, strong interfacial interactions, and structural stability. However, their highly heterogeneous and irregular polymer architecture, together with limited solubility and processability, restricts their direct use as a tunable building block compared with lower‐molecular‐weight polyphenols [64, 65]. Thus, lignins are more commonly explored as a reinforcing or functional additive rather than a primary structural motif in polyphenol‐based materials for agriculture.

### 2.2 Core Features Enabling Polyphenol Functions

The abundant phenolic groups in polyphenols enable interactions with diverse materials and substrates through noncovalent interactions (e.g., metal coordination, π interactions, hydrogen bonding, and electrostatic and hydrophobic interactions), as well as covalent interactions (e.g., formaldehyde‐induced polymerization, boronate–phenolic network complexation, silicon chemistry, Michael addition and Schiff‐base reactions, thiyl radical oxidation, Mitsunobu reactions, acylation, and epoxy chemistry) (Figure 3). Among the abovementioned noncovalent interactions, metal coordination is one of the most widely employed strategies for engineering multifunctional polyphenol‐based materials (e.g., metal–phenolic network (MPN) composites) [12]. This interaction arises from the coordination of metal ions with the electron‐donating oxygen atoms of phenolic hydroxyl groups, where the lone pairs on these oxygen atoms are donated to vacant orbitals of metal ions [16]. Polyphenol‐mediated covalent interactions involve the formation of shared‐electron bonds between phenolic moieties (or their oxidized derivatives) and complementary reactive motifs. These covalent linkages generally endow greater structural stability than noncovalent interactions, making them of interest for applications that require prolonged performance under complex or harsh environmental conditions (e.g., agricultural systems) [66]. The coexistence of noncovalent and covalent interactions affords versatile tunability of polyphenol‐enabled materials, providing the physicochemical foundation for the structural adaptability and multifunctionality of these materials, as discussed below.

**FIGURE 3:**
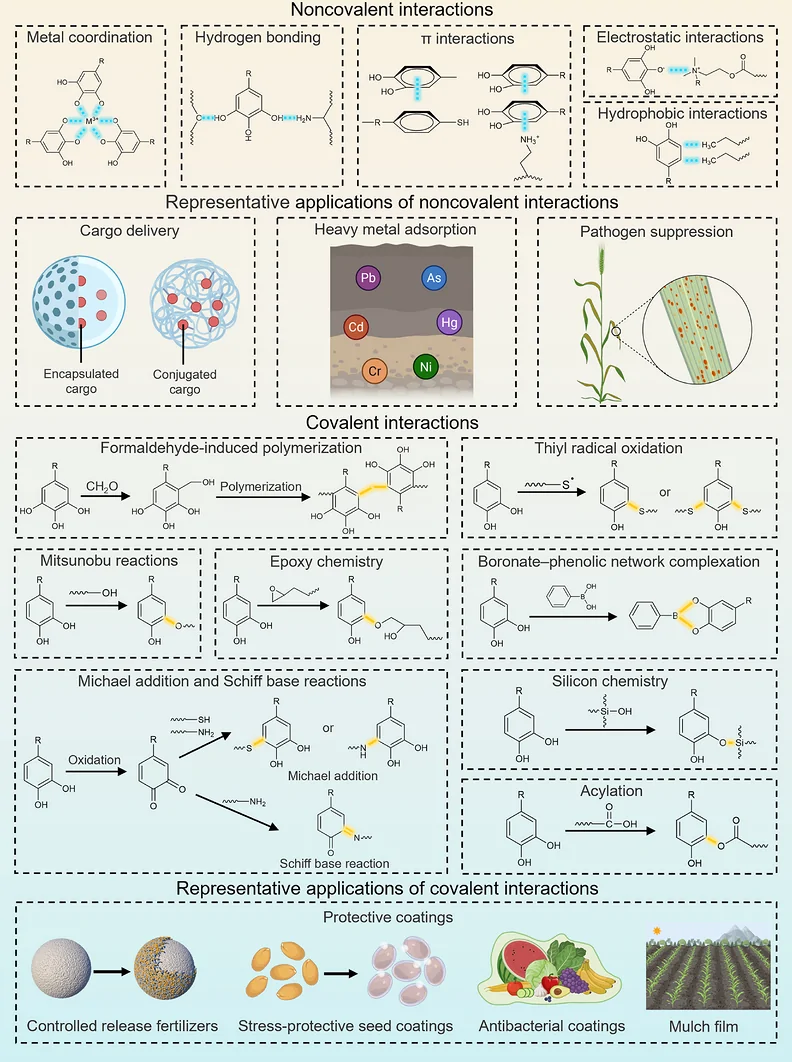
Polyphenol‐mediated noncovalent and covalent interactions and representative applications. Created in BioRender.com. Liu, H. (2026) https://BioRender.com/50su11q.

#### 2.2.1 Adhesion Properties

Adhesion is one of the most important requirements for coating applications in agriculture, specifically in pesticide formulations and seed treatments, as it determines the retention of coatings under rainfall, durability during application, and persistence in soil. Conventional polymer adhesives (e.g., cyanoacrylates and epoxies) fail in wet environments and are toxic and nondegradable [67]. In contrast, polyphenols possess catechol and galloyl groups that enable multiple bonding modes with diverse substrates, offering a sustainable route to strong and versatile adhesion [68]. The adhesion of polyphenols derives from a combination of noncovalent (e.g., hydrogen bonding, π–π stacking, and hydrophobic interactions) and covalent interactions (e.g., via Michael addition and Schiff base reactions, boronate–phenolic network complexation, and triyl radical oxidation), supporting interfacial anchoring (Figure 3). For example, quinone‐mediated Schiff base and Michael addition reactions establish covalent links with nucleophiles such as amines and thiols [69].

The adhesion performance of polyphenols on various substrates is due to their rich interfacial interactions with substrates and adaptable functionalization. Polyphenols can interact with amine and epoxy functional groups, and polymer backbones, enabling multivalent bonding through hydrogen bonding, covalent interactions, π–π stacking, and coordination, thereby enhancing interfacial adhesion under diverse environmental conditions [70]. Zhang et al. demonstrated the epoxidation of polyphenols to develop hydrogels with strong adhesion properties on various substrates under both dry and humid conditions, while maintaining resistance to solvents and low temperatures [71]. Diverse adhesive architectures, including hydrogels, polyphenol–polymer composites, and MPNs provide tunable mechanical compliance, cohesive strength, and interfacial robustness [72, 73]. A representative hydrogel system fabricated from tannic acid (TA), hyperbranched polylysine, and acrylic acid via three‐dimensional (3D) printing enables precise, reproducible, and customizable adhesive design [73]. In another study, ionic liquid microcapsules stabilized by MPNs exhibit strong interfacial adhesion and penetration across biofilms, enabling rapid clearance of planktonic, biofilm‐embedded, and intracellular bacteria while promoting infected wound healing without antibiotics [74]. Furthermore, the versatility of polyphenol‐mediated adhesion extends across chemically and physically different substrates, such as glass and ceramics, metals, polymers with low surface energy, biological tissues, and natural materials such as wood. As exemplified by bioinspired gelatin–TA hydrogels, strong and durable adhesion can be achieved even on wet and chemically inert surfaces, highlighting the capacity of polyphenols to bridge interfaces through diverse interactions [75].

Building on the strong interfacial adhesion of polyphenols, polyphenol‐enabled systems have been widely applied in agriculture. For example, polydopamine (PDA)‐coated avermectin microcapsules have shown to prolong the retention of the pesticide on corn and cotton leaves, while simultaneously protecting the pesticide from ultraviolet (UV) degradation and improving field stability [76]. Likewise, lignin–polyphenol nanocolloids enhance the adhesion and persistence of fungicides, reducing wash‐off losses under rainfall and slowing photodegradation [77]. Other studies combine adhesion with protective functions. PDA–graphene oxide hybrids improve leaf‐surface attachment while enabling photothermal activation [78], and cellulose nanocrystal‐stabilized PDA microcapsules provide strong interfacial anchoring together with UV shielding and high pesticide encapsulation efficiency [79]. Beyond crop protection, PDA surface‐functionalized Fe_3_O_4_ nanoparticles demonstrate high affinity for insect guts and trigger localized heating upon near‐infrared irradiation, potentially enabling pest control without the use of chemical pesticides [80].

#### 2.2.2 Synthesis Strategies for Polyphenol‐Enabled Material Platforms

Sustainable agricultural applications require materials that can be produced under mild and environmentally friendly conditions. Many advanced materials (e.g., polymers, inorganic nanostructures, and hybrid composites) have shown excellent performance in controlled release and crop protection. However, their fabrication typically requires high temperatures, organic solvents, or toxic catalysts, which complicate scale‐up and raise concerns about environmental compatibility [81]. In contrast, polyphenol‐based materials can be synthesized through simple, aqueous, and catalyst‐free processes, while still providing tunable structures and versatile functionalities. Such synthesis characteristics not only minimize energy and chemical consumption but also provide potential for applications such as nutrient and pesticide delivery.

Self‐assembly of polyphenols in aqueous phases provides a sustainable and versatile fabrication pathway consistent with green and environmentally friendly principles in agriculture. For example, water‐soluble pesticides (e.g., myclobutanil) have been delivered through their complexation with TA in water. This assembly strategy enables the pH‐responsive release of pesticides, enhances rain fastness, and improves antifungal efficacy, as well as reducing the genotoxicity of myclobutanil to plant cells [82]. Similarly, lignin–tannin nanoparticles can be formed in aqueous systems as natural UV‐shielding additives, stabilizing photosensitive pesticides and protecting active molecules from photodegradation, while maintaining visible light transmittance [83]. Tea polyphenol–Zn^2+^ nanocomposite coatings have demonstrated multifunctionality in aqueous phases, combining antioxidant, antibacterial, and moisture‐retention properties to significantly prolong the shelf life of fruits in postharvest chains (Figure 4a) [84]. Beyond crop protection, TA–Fe^3+^ coordination has been used for the rapid aqueous deposition of MPNs on biomass surfaces, resulting in low‐cost adsorbents and demonstrating the scalability of water‐based self‐assembly [85].

**FIGURE 4:**
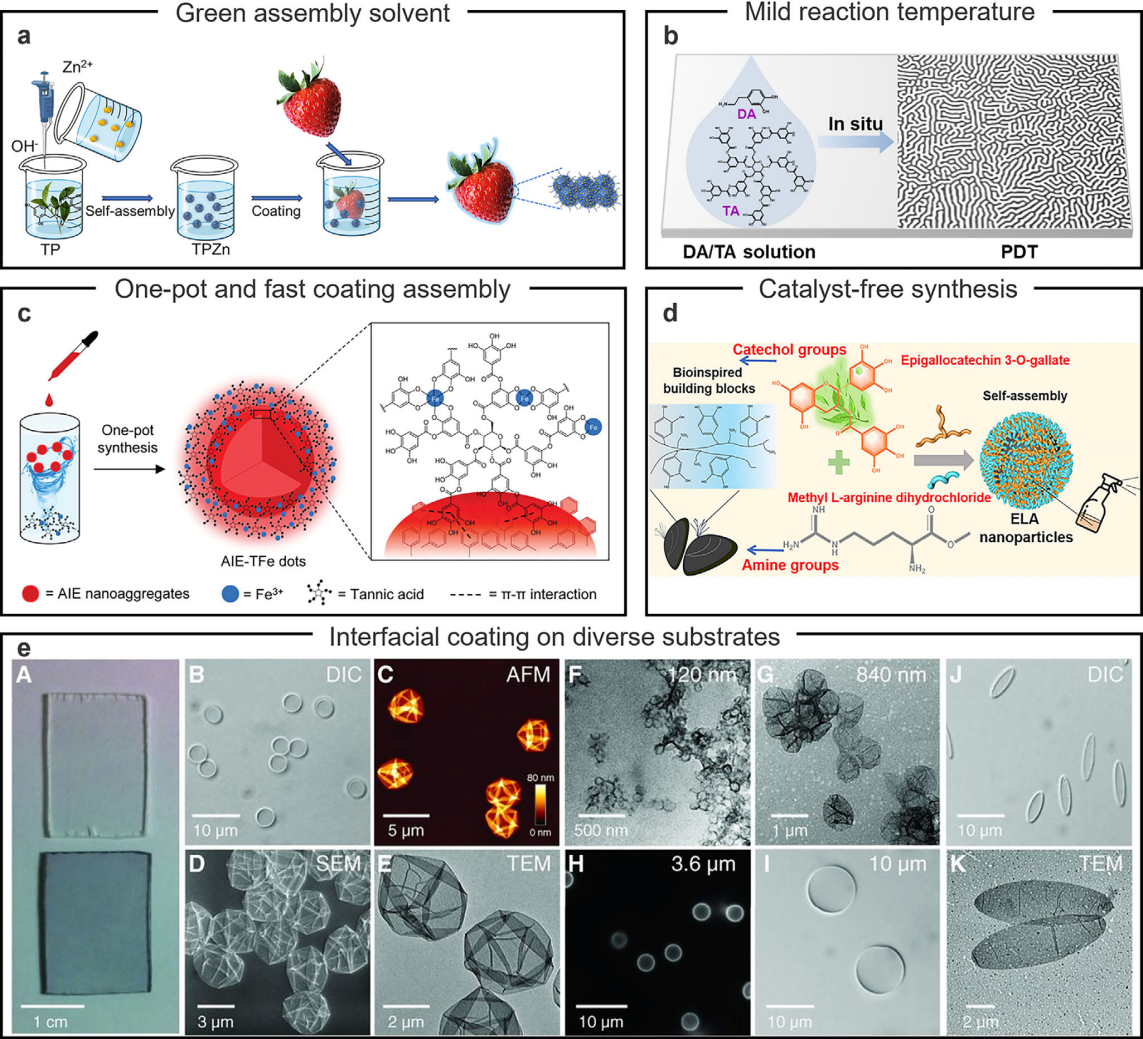
Synthesis strategies of polyphenol‐enabled material platforms for agricultural applications. (a) Preparation of a tea polyphenol–Zn^2+^ nanocomposite (TPZn) coating on fruits for fruit preservation. Adapted with permission [84]. Copyright 2025, American Chemical Society. (b) Schematic illustration of the rapid in situ fabrication of polydopamine–TA nanocoating (PDT) through polyphenol chemistry. DA, dopamine. Adapted with permission [86]. Copyright 2025, Wiley‐VCH GmbH. (c) Schematic of one‐pot synthesis of aggregation‐induced MPN‐coated emission luminogens (AIEgens). Adapted with permission [90]. Copyright 2022, Xu et al., published by Wiley‐VCH GmbH. (d) Schematic of the catalyst‐free assembly of EGCG and l‐arginine (to form ELA) as antibacterial coatings with anti‐water washing and antioxidant properties. Adapted with permission [92]. Copyright 2025, American Chemical Society. (e) Fe^3+^–TA films prepared on various substrates of different shapes (planar, spherical, and ellipsoidal) and sizes (120 nm to 10 µm). Photograph of polystyrene slides before (top) and after (bottom) Fe^3+^–TA coating (e‐A). Microscopy images of Fe^3+^–TA capsules: differential interference contrast (DIC) images (e‐B, e‐I, and e‐J); atomic force microscopy (AFM) images (e‐C); scanning electron microscopy (SEM) image (e‐D); transmission electron microscopy (TEM) images (e‐E, e‐F, e‐G, and e‐K); and fluorescence microscopy image (e‐H). Adapted with permission [72]. Copyright 2013, American Association for the Advancement of Science.

Polyphenols can be deposited onto diverse substrates at room temperature, further reducing energy costs during manufacturing. PDA–polyphenol surface engineering enables the formation of nanocoatings within a few minutes under neutral conditions (Figure 4b), creating hierarchical roughness, and enhancing interfacial adhesion, wet stability, and cargo loading without requiring external heating [86]. Moreover, the enzymatic pathways provide an alternative approach: laccase‐triggered co‐deposition of gentisic acid and chitosan proceeds at room temperature and under mild oxidation potential, yielding uniform antimicrobial films with controllable thickness growth and durable performance [87]. Polyphenol chemistry has also been harnessed to induce strong adhesion of liquid metal inks on diverse substrates, enabling patternable conductive tracks at room temperature [88], which are suitable for low‐temperature processing of agricultural devices and packaging. Furthermore, bioinspired adhesives that integrate catechol/galloyl motifs with protein‐mimetic sequences achieve repeatable and water‐resistant adhesion at room temperature. Such adhesives provide a model for seed coatings, sensor integration, and field‐deployable repairs where mild temperatures are required for applications [89].

One‐pot and in situ strategies have helped simplify the synthetic route for polyphenol‐based materials and reduce costs. For example, the one‐pot synthesis of MPN‐coated nanoparticles has been extended to biomedical systems, where hydrophobic small molecules are encapsulated with enhanced resistance to harsh environments (e.g., pH, oxidation, and temperature) (Figure 4c) [90], which is relevant for agricultural applications that require the protection of labile actives. Chitosan/poly(vinyl alcohol) matrices have been functionalized via a one‐pot process using polyphenols as both reducing and cross‐linking agents to yield nanocomposites with a uniform particle distribution and improved mechanical stability. When used for meat preservation, the modified films extended the shelf life of lamb meat at 4°C, maintaining quality for up to 30 days [91].

Catalyst‐free assembly represents a key green synthesis strategy for polyphenol‐enabled materials, where noncovalent interactions such as hydrogen bonding, coordination, and redox reactions drive material formation without external catalysts. For example, Du et al. have reported that EGCG and amino acid surfactants (e.g., l‐arginine) can spontaneously assemble into multifunctional coatings in water without the need for catalysts, producing films that retain antimicrobial activity even after repeated washing (Figure 4d) [92]. Most reported assembly technologies (e.g., TA–metal coordination and lignin‐based carriers) can be constructed without external catalysts, highlighting the general applicability of catalyst‐free synthesis strategies [83, 87, 93, 94]. A metal–polyphenol shell has been fabricated in the absence of a catalyst to enable the controlled release of enzymes (e.g., hyaluronidase) in solid tumor treatment [95], and rough PDA–polyphenol nanocoatings have been developed by exploiting redox chemistry without additional reagents to exhibit antioxidant properties and high dye adsorption capacities [86].

Metal–ligand complexation represents a fundamental synthesis strategy in polyphenol‐based materials, where coordination between phenolic groups (e.g., catechol or galloyl) and metal ions drives rapid interfacial assembly. This coordination process enables the formation of conformal coatings across diverse substrates. Pioneering work by Ejima et al. demonstrated that polyphenol–metal coordination (to form MPNs) enables conformal coating of planar surfaces, spherical objects, and even microorganisms, highlighting the interfacial versatility of this approach (Figure 4e) [72]. The multiple interactions and functionalities of polyphenols provide a modular basis for further functionalization, thereby greatly expanding the design scope and application of MPN‐based systems [96]. Recent studies have expanded MPN coatings from substrate‐independent deposition to function‐directed interfacial engineering. For example, MPN interlayers have been used to grow crystalline metal–organic framework (MOF) coatings on both particle and planar substrates, with tunable sizes, shapes, and surface chemistries, highlighting their role in regulating secondary material growth [97]. Moreover, Fe^3+^‐TA single‐cell nanocoatings have been developed as probiotic “nanoarmor,” protecting both Gram‐positive and Gram‐negative bacteria against multiple antibiotics [98].

#### 2.2.3 Polyphenol‐Mediated Cargo Encapsulation

Encapsulation represents a fundamental feature of polyphenol‐based assemblies, defining how diverse cargos (active ingredients) are loaded and protected before their release in the agricultural field. Polyphenol‐mediated encapsulation exploits multivalent coordination and interactions, enabling the integration of small molecules, metal ions, proteins, nucleic acids, and biological entities within the assembled structure. The selective permeability of these assemblies enables the gating and immobilization of cargos according to their size and chemical properties, while dynamic interactions afford controlled cargo release in response to environmental stimuli such as pH, redox conditions, and enzymatic activity [99].

TA–Fe^3+^‐coated Pickering emulsions function as smart microcapsules that integrate cargo (e.g., imidacloprid) protection with interfacial adhesion and UV resistance [100]. Polyphenol‐mediated nanoparticles enable the encapsulation of bioactive molecules (e.g., silicon ions, interferon alpha, and nucleic acids), supporting antioxidant activity, gene delivery, and glucose metabolism regulation [101, 102, 103]. These polyphenol‐enabled nanocarriers highlight their potential for the delivery of nutrients, pesticides, and phytohormones in agriculture. Furthermore, polyphenol‐mediated materials have been extended to vesicular and cell‐derived systems, including exosome delivery systems, anti‐oxidase complexes, and cell‐mimetic nanosponges, where encapsulation couples high payload capacity with targeting and environmental protection [104, 105, 106, 107]. Other studies have established polyphenol‐mediated encapsulation mechanisms, particularly the roles of programmable permeability [26, 108], guest competition, and coordination‐driven reconfiguration, as well as their integration with catalytic or cascade therapeutic functions [109, 110, 111]. Thus, polyphenol‐mediated cargo encapsulation strategies can enable programmable release, high transport efficiency, and environmental responsiveness at the plant–soil interface.

Specifically, the same encapsulation principles are increasingly applied to polyphenol technologies in agricultural systems, where cargo stability, environmental robustness, and controlled release are essential. MPN nanoparticles have been employed to encapsulate pesticides, improving foliar retention, UV resistance, and field efficacy while reducing off‐target losses [112]. A similar polyphenol‐mediated encapsulation strategy has been applied in food preservation, for example in konjac glucomannan‐based films incorporating polyphenol–metal nanoparticles [113]. Moreover, polyphenol‐based nanocoatings have enabled the encapsulation and protection of living cargos, such as nitrogen‐fixing bacteria, allowing stable foliar colonization and sustained biological activity under outdoor conditions [114]. Silicate–phenolic networks (SPNs) afford encapsulation of highly soluble nutrients through coordination confinement, enabling the controlled release of fertilizers [115]. These advances collectively demonstrate that polyphenol‐enabled encapsulation provides a materials framework that combines precise cargo control with the practical demands of agricultural delivery systems.

#### 2.2.4 Tunable Functionalities of Polyphenol Assemblies

Polyphenol assemblies not only act as carriers but their dynamic interactions with encapsulated cargo enable a transition from diffusion‐dominated release toward stimulus‐responsive delivery behaviors [116]. Through the selection of polyphenols, guest molecules, and assembly conditions, both interfacial interactions and stimuli‐responsiveness of the resulting assemblies can be systematically tuned. This allows polyphenol assemblies to respond to specific environmental or biological stimuli (e.g., pH, temperature, glucose, and enzyme) rather than humidity and pressure from soil. In contrast to conventional release processes governed primarily by moisture penetration and mechanical disruption, polyphenol‐based assemblies enable active release in response to pH variations, temperature fluctuations, enzymatic activity, and metabolite availability within the rhizosphere or plant microenvironment (Figure 5). For instance, pH‐responsive TA–Fe^3+^ coatings on cellulose nanocrystal emulsions provide bi‐directional pesticide release, while ensuring foliar adhesion and UV stability [100]. Thermo‐switchable Janus hydrogels extend this concept to polymer networks, where PDA‐mediated adhesion changes over three orders of magnitude across physiological temperatures, allowing on‐demand detachment and drug release [117]. Enzyme‐mediated polyphenol coacervates demonstrate protease‐specific release while maintaining colloidal integrity in complex biological environments, underscoring the capacity of polyphenol assemblies to couple biochemical recognition with controlled delivery [118]. The flexibility in MPNs highlights this principle, as coordination density and guest competition mediate reversible permeability and metabolite‐responsive disassembly [26].

**FIGURE 5:**
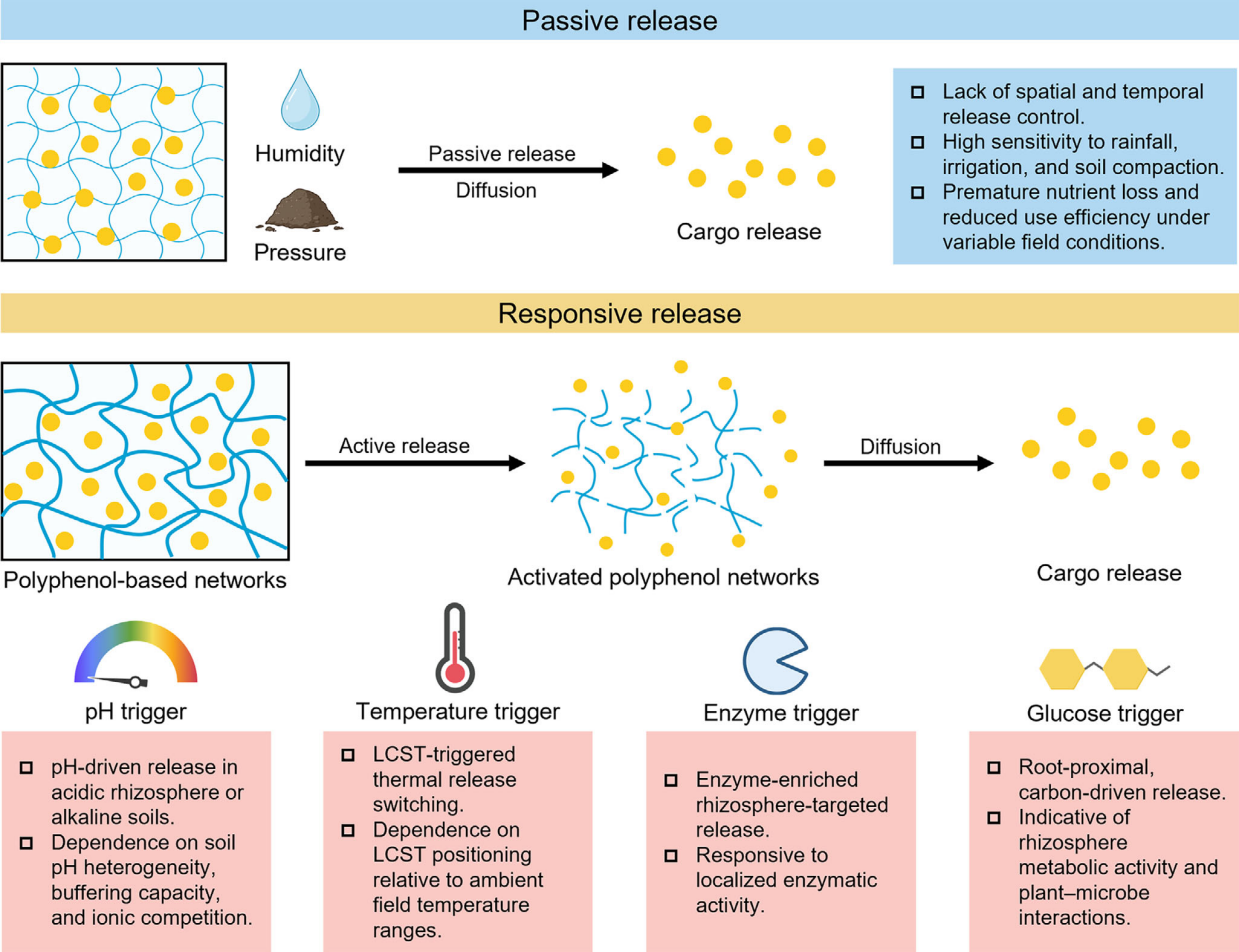
From passive release to responsive release in polyphenol‐based agricultural systems. Conventional release (i.e., passive release) relies primarily on moisture penetration and mechanical disruption, resulting in passive diffusion that lacks spatial and temporal control under heterogeneous field conditions. In contrast, polyphenol‐based systems leverage dynamic coordination chemistry and multivalent interactions to enable active, environment‐dependent release in response to localized pH variations, temperature fluctuations, enzymatic activity, and metabolite availability within the rhizosphere or plant microenvironment (i.e., responsive release). By coupling release behavior to environmental and biological triggers rather than bulk exposure, polyphenol assemblies provide a tunable platform for adaptive agrochemical delivery. LCST, lower critical solution temperature. Created in BioRender.com. Liu, H. (2026) https://BioRender.com/teklkj8.

Polyphenol assemblies serve as multifunctional platforms, enabling the integration of intrinsic redox catalysis, antimicrobial activity, biosensing, solar desalination, imaging, and photoprotection (Figure 6). The radical‐scavenging ability of phenolic groups provides continuous reactive oxygen species (ROS) buffering, while coordination with bioactive ions (e.g., Mg^2+^) strengthens network stability [119]. Pathogens and extreme environmental conditions are major factors that limit plant growth. This redox activity is particularly important for alleviating oxidative stress in plants and enhancing their ability to survive under adverse conditions. The interaction between polyphenols and various metal ions facilitates the transport of micronutrients within plants. MPNs can enhance antimicrobial activity through coordinated interactions, leading to effective disruption of microbial membranes and inhibition of pathogen growth [120], which can be leveraged to suppress plant pathogens and regulate microbial populations in the rhizosphere and on plant surfaces. Polyphenol‐based systems enable responsive biosensing through reversible phenolic bonding, allowing detection of environmental signals such as pH and ionic changes. For example, acid‐sensitive fabrics built from TA–amino acid linkages exhibit reversible pH‐dependent color changes, enabling real‐time environmental detection [121]. Directional MPNs engineered for solar desalination combine strong light absorption with salt‐rejecting channels, allowing continuous freshwater production while suppressing membrane fouling under high salinity [122]. This strategy can potentially provide low‐energy freshwater sources for irrigation in saline or water‐poor environments, while mitigating salt accumulation and membrane fouling. Polyphenol assemblies can be engineered as tunable imaging platforms, where controlled coordination architectures convert phenolic group amplification into predictable signal enhancements [123]. This makes it possible to track the transport and distribution of nanomaterials in plants and soil, which is essential for the increasing use of nanomaterials in agricultural cargo delivery. MPN‐coated zinc oxide nanoparticles provide effective photoprotection by suppressing photo‐induced reactivity at material interfaces. Through spontaneous coordination and controlled oxidation with zinc oxide, polyphenols broaden UV absorption and attenuate photocatalytic activity, thereby stabilizing photoactive substrates under prolonged light exposure [124].

**FIGURE 6:**
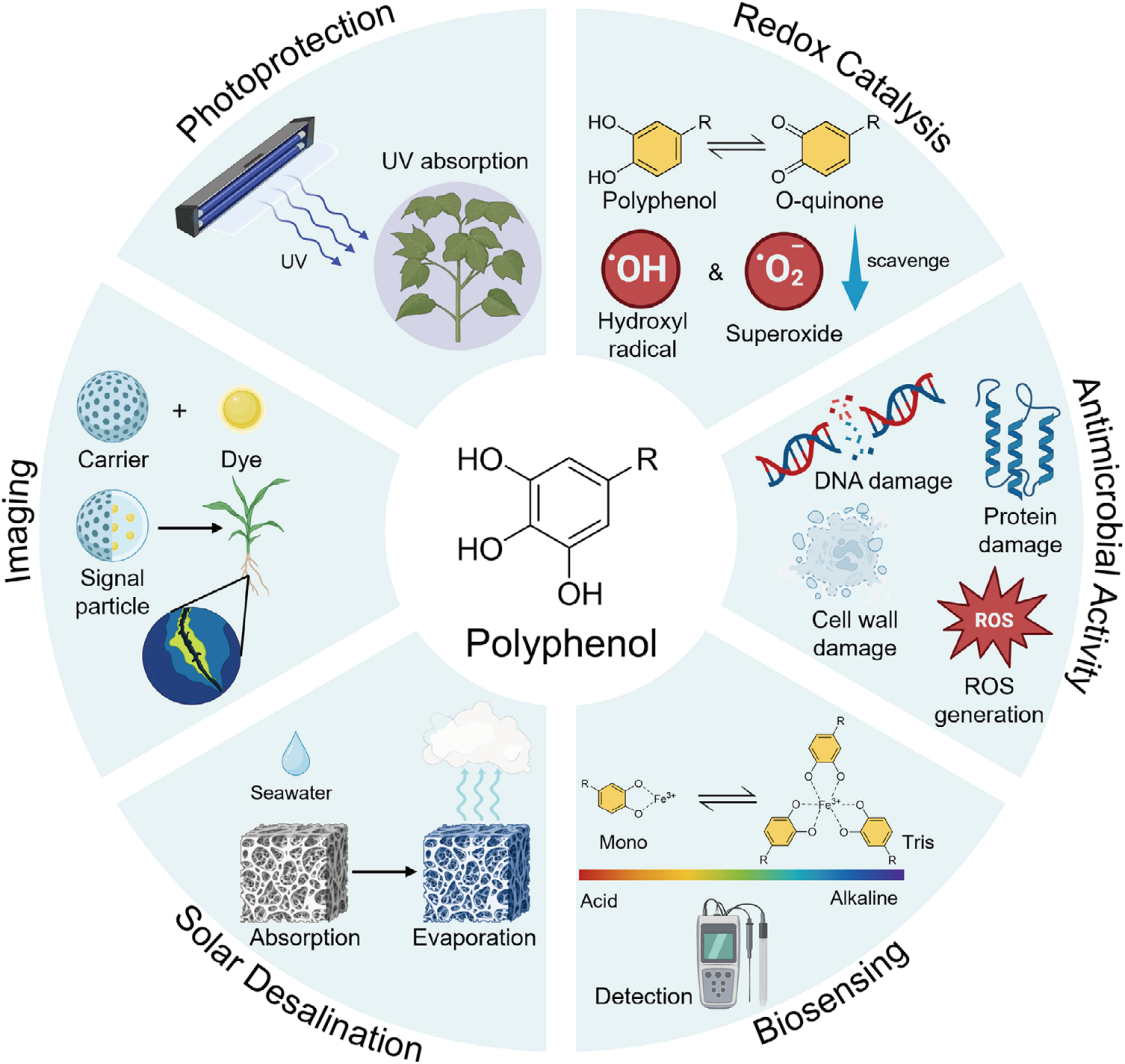
Diverse and tunable functionalities of polyphenol assemblies. Created in BioRender.com. Liu, H. (2026) https://BioRender.com/899zgs4.

Polyphenols can be engineered into diverse assembly platforms, including films/coatings, carriers, and hydrogels. Therefore, the selection of platform should be determined by the intended agricultural scenario, considering the physicochemical properties of the cargo, the interfacial requirements, and the targeted release kinetics (Table 1).

**TABLE 1:** Comparison of agricultural scenarios, opportunities, and limitations of different polyphenol‐enabled assembly platforms for agricultural applications.

Assembly platform	Agricultural scenario	Opportunities	Limitations
Films/coatings	Coating leaves, seeds, fertilizer granules, fruits, soil particles, or microbial surfaces that require adhesion, protection, or diffusion‐barrier control	Broad substrate adhesion; flexible deposition approaches; tunable coating thickness	Limited long‐term durability; limited suitability for hydrophobic applications
Carriers (e.g., particles, capsules, and conjugates)	Delivery of pesticides, nutrients, bio‐stimulants, or other cargos that require nanoscale dispersion, encapsulation, photoprotection, or controlled release	Tunable particle size and surface chemistry; improved cargo loading; enhanced dispersion, photostability, and release control	Possible particle aggregation
Hydrogels	Soil water retention and rhizosphere cargo delivery that require water‐holding capacity, swelling control, and long‐term stability	Tunable cross‐linking density; adjustable permeability and swelling behavior; improved mechanical integrity; long‐term release capability	Diffusion‐limited release; potential requirement for toxic cross‐linkers or curing

## 3 Applications of Polyphenol‐Inspired Material Platforms in Agriculture

The features and functionalities of polyphenols dictate their applications in agriculture. This section reviews the diverse agricultural applications of polyphenol‐enabled material platforms, including soil remediation, seed coating, controlled release of nutrients, pesticide delivery, sensing and monitoring, nitrification inhibition, and food preservation and shelf‐life extension. A comparison of representative polyphenol‐enabled systems [112, 113, 114, 115, 125, 126, 127, 128, 129, 130, 131, 132, 133, 134, 135, 136, 137, 138, 139, 140, 141, 142, 143, 144, 145, 146, 147, 148, 149, 150, 151, 152, 153, 154, 155, 156, 157, 158, 159, 160, 161, 162, 163, 164, 165, 166] currently employed in agricultural applications is summarized in Table 2, highlighting their diversity and features.

**TABLE 2:** Overview of polyphenol‐based material platforms across major agricultural applications.

Agricultural applications	Material platforms	Features	Cross‐linking methods	Refs.
Soil remediation	Polyphenol‐functionalized biochar/carbon materials	Surface functional group enrichment; cation‐exchange capacity enhancement; pH buffering capability; redox‐active surface modulation	Metal coordination; hydrogen bonding; π interactions; electrostatic interactions; hydrophobic interactions	[125, 126, 127, 128]
	Polyphenol‐based hydrogels	High adsorption capacity; water‐retention capacity modulation; tunable porosity	Metal coordination; hydrogen bonding; π interactions	[129, 130, 131, 132]
Seed coating	Polyphenol–metal networks	Shell thickness and compactness control; surface adhesion enhancement	Metal coordination; hydrogen bonding; π interactions; electrostatic interactions	[112, 133, 134]
	Polyphenol–hydrogel composites	Water‐retention capacity modulation; controlled release of bioactive agents; redox‐active additive incorporation	Hydrogen bonding; electrostatic interactions	[135, 136]
Controlled nutrient release	Polyphenol–metal coordination shells	Coordination density and shell compactness control; nonaqueous and solvent‐free assembly compatibility; environmental responsiveness	Metal coordination; hydrogen bonding; π interactions; electrostatic interactions	[114, 137, 138, 139]
	Polyphenol–inorganic hybrids (e.g., silicate–phenolic networks)	Hybrid network densification; coating thickness control; water‐permeation barrier adjustment	Silicon chemistry	[115]
	Polyphenol–polymer composites (e.g., TA–PVA, TA–alkylamine, poly(TA))	Robust cross‐link networks; prolonged release profiles	Hydrogen bonding; Michael addition and Schiff base reactions	[140, 141, 142, 143]
Pesticide delivery	Polyphenol–metal networks (e.g., TA–Fe^3+^, catechol–Cu^2+^)	Dynamic coordination bonds; pH/redox/enzyme‐responsive disassembly	Metal coordination; hydrogen bonding; π interactions; electrostatic interactions	[144, 145, 146, 147]
	Polyphenol–polymer composites (e.g., TA–rosin)	Cross‐link density control; programmable permeability; enhanced mechanical robustness	Metal coordination; hydrogen bonding; π interactions	[148]
	Polyphenol–porous inorganic hybrids (e.g., TA–MOF, TA–ZIF)	Tunable pore size and specific surface area; adjustable pore volume and loading capacity	Metal coordination	[149, 150, 151]
Sensing and monitoring	Polyphenol–metal networks	Metal–ligand coordination dynamics; redox‐responsive optical modulation; film thickness and surface uniformity control	Metal coordination; hydrogen bonding; π interactions; electrostatic interactions	[152, 153, 154, 155, 156]
	Polyphenol‐based nanozymes	Active site density regulation; catalytic activity modulation	Metal coordination	[157]
Nitrification inhibition	Polyphenol–metal networks	Metal–ligand coordination strength; redox‐active phenolic group density; enzyme‐interaction capability	Metal coordination; hydrogen bonding; π interactions; electrostatic interactions	[158, 159]
	Polyphenol–inorganic hybrids	Encapsulation stability control; controlled inhibitor release	Hydrogen bonding	[160]
Food preservation	Polyphenol‐based hydrogels	Water‐retention capacity modulation; surface adhesion and film‐forming ability	Metal coordination; hydrogen bonding; electrostatic interactions; boronate–phenolic network complexation	[161, 162, 163]
	Polyphenol‐based nanoparticles	Particle size and surface charge control; functional regulation with different metals	Metal coordination; hydrogen bonding; π interactions; electrostatic interactions	[113, 164, 165]
	Polyphenol‐based nanocrystals	Crystal size and morphology regulation; surface functional group density	Metal coordination; hydrogen bonding	[166]

Abbreviations: MOF, metal–organic framework; PVA, poly(vinyl alcohol); TA, tannic acid; ZIF, zeolitic imidazolate framework.

### 3.1 Polyphenol‐Mediated Soil Remediation

Soil pollution caused by heavy metals, microplastics, and aromatic dyes poses persistent risks to agricultural productivity and ecosystem health. Polyphenol‐enabled materials not only immobilize toxic ions but also mitigate the impact of plastic residues and capture organic dyes through π interactions or hydrogen bonding (Table 2).

#### 3.1.1 Heavy Metal Remediation

Polyphenol‐based systems have reshaped conventional approaches to heavy metal immobilization. Polyphenol‐functionalized carbon materials have proven effective in immobilizing heavy metals, while improving soil quality. For example, lignin‐derived biochar (LBC) enriched with phenolic and carboxylic sites promotes the conversion of Cd^2+^ from exchangeable to residual forms, achieving immobilization efficiencies exceeding 90%, while enhancing soil pH, cation‐exchange capacity, and microbial activity. The coordination of metal ions with hydroxyl groups in lignins stabilizes toxic species and facilitates long‐term sequestration within the carbon matrix (Figure 7a) [128]. Moreover, in Cd^2+^/Zn^2+^ co‐contaminated soils, LBC forms stable minerals with soil, enhancing its capacity to adsorb Cd^2+^ and Zn^2+^. The gradual release of alkaline ions (Si^4+^, Mg^2+^) raises soil pH, thus increasing the activity of antioxidant enzymes such as catalase and superoxide dismutase [126]. This coupling of chemical stabilization with microbial resilience highlights that polyphenol‐modified materials can support soil remediation not only by immobilizing contaminants, but also by preserving or restoring biological activity. Recently, Santorufo et al. have demonstrated that TA‐modified biochar exhibits a higher abundance of hydroxyl groups than unmodified biochar, resulting in enhanced soil enzyme activity. This study further demonstrates that polyphenol‐mediated surface modification can promote soil remediation through both chemical and biological pathways [125]. Moreover, the strong interaction between TA and Fe^3+^ oxides, as described by Kaal et al., leads to the formation of robust Fe─O─phenol coordination that can remain stable under a wide range of soil conditions [167]. TA can also promote arsenic mobilization through the decomposition of Fe─As mineral phases, followed by the complexation of released Fe^3+^ ions, which prevents re‐precipitation and facilitates the release of arsenic into solution [168]. Overall, polyphenols and polyphenol‐based modifiers provide a viable pathway for heavy metal remediation. Through pH buffering, coordination‐driven precipitation, and microbiome modulation, these materials translate molecular chelation into practical strategies for soil detoxification. We note that although polyphenols are generally considered biodegradable and nontoxic, the effects of their large‐scale application in the field on plant growth, soil microbial communities, and potential toxicity require further investigation.

**FIGURE 7:**
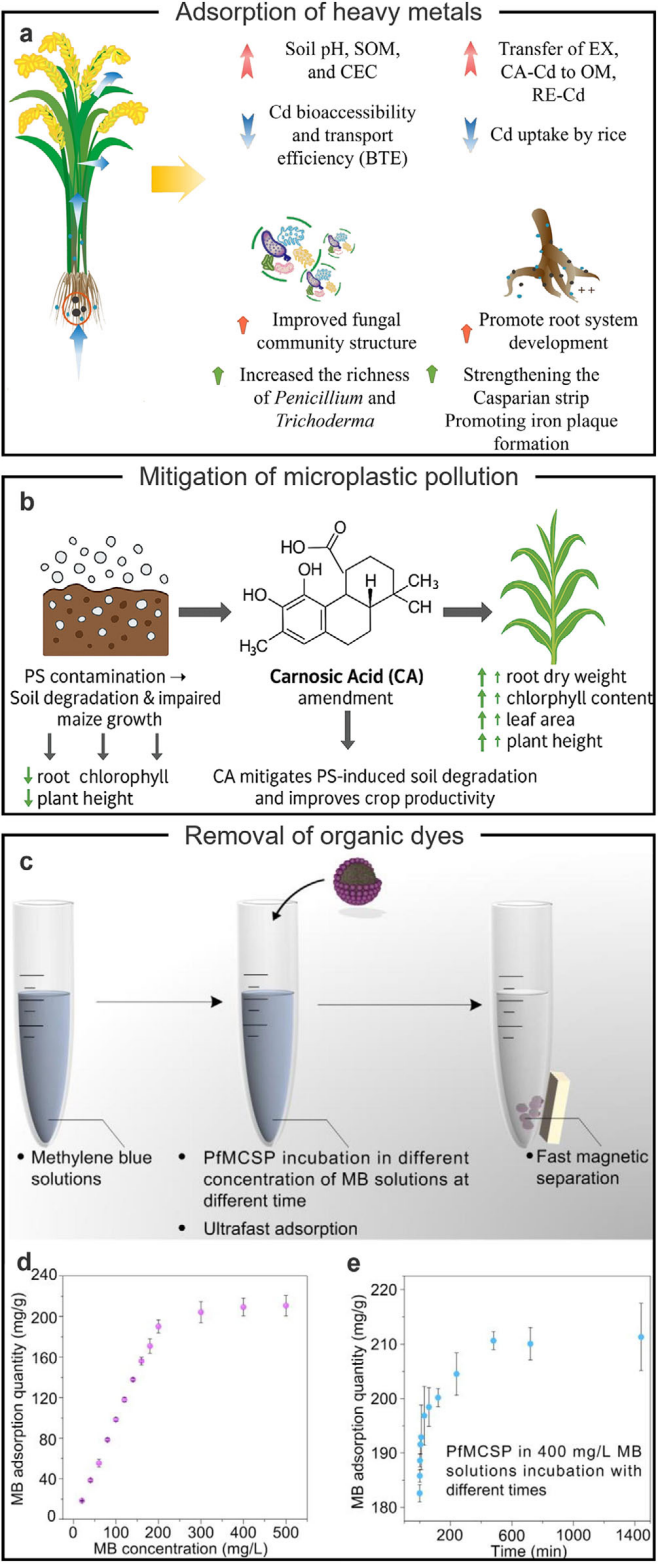
Polyphenol‐based materials for soil remediation. (a) Schematic diagram of a soil remediation strategy coupled with safe rice production using LBC. SOM, organic matter content; CEC, cation exchange capacity; EX, exchangeable fraction; CA–Cd, carbonate‐bound fraction of cadmium; OM, Fe/Mn oxide‐bound fraction; RE‐Cd, residual fraction of Cd. Adapted with permission [128]. Copyright 2024, Elsevier B.V. (b) Schematic showing the alleviation of polystyrene (PS) microplastic‐induced soil degradation and enhancement of maize growth using carnosic acid. Adapted with permission [170]. Copyright 2026, Taylor & Francis Group, LLC. (c) Schematic of methylene blue (MB) dye removal using polyphenol‐functionalized magnetic core–satellite superstructure (PfMCSP). (d) MB adsorption amount of PfMCSP as a function of MB concentration (20–500 mg L^−1^) and (e) time (0–1400 min) at an MB solution concentration of 400 mg L^−1^. (c)–(e) Adapted with permission [171]. Copyright 2022, Elsevier B.V.

#### 3.1.2 Polyphenol‐Enabled Strategies for Mitigating Microplastic Pollution

The accumulation of microplastics in soil disrupts structure, nutrient exchange, and microbial ecology [169]. Polyphenols can interact with microplastics via noncovalent interactions (e.g., π–π stacking, hydrophobic interactions, and hydrogen bonding), leading to an increase in the degradation of plastics through catalytic activities. Polyphenol‐derived small molecules can actively mitigate microplastic‐induced soil degradation by modulating soil biochemical processes rather than relying on physical removal. For example, carnosic acid, a naturally occurring phenolic diterpene, has been shown to alleviate polystyrene microplastic‐induced soil deterioration by restoring soil enzymatic activities, reducing oxidative stress, and improving nutrient availability [170]. Through its strong antioxidant capacity and redox‐active phenolic structure, carnosic acid suppresses microplastic‐triggered ROS accumulation in soil–plant systems, thereby preserving root function and promoting maize growth under microplastic‐contaminated conditions. The study highlights a complementary polyphenol‐enabled remediation pathway, in which chemical buffering and biological regulation, rather than particle capture, are exploited to mitigate the ecological risks of microplastic pollution in soil (Figure 7b) [170].

#### 3.1.3 Organic Dye Removal

Polyphenol‐functionalized materials have also demonstrated potential for adsorbing and eliminating organic dyes and aromatic pollutants in soil and aqueous environments. Jin et al. have demonstrated the functionalization of polyphenols to induce hierarchical aggregation within magnetic superstructures, generating abundant accessible pores and conjugated π‐domains that facilitate the adsorption and electron transfer of organic dyes (Figure 7c). The Fe_3_O_4_@TA–Fe^3+^ networks achieve rapid and selective uptake of organic dyes (e.g., methylene blue, rhodamine B, and toluidine blue) with magnetic recovery enabling reuse over multiple cycles, while retaining more than 95% of the original capacity (Figure 7d,e) [171]. Subsequent developments have expanded this concept toward structure–function modulation. For example, Tang et al. reported a porous biochar obtained via pyrolysis of polyphenol and metal ions co‐functionalized collagen. This strategy improved pore connectivity and redox activity, facilitating the removal of antibiotics, heavy metal ions, and dyes [172].

### 3.2 Polyphenol‐Mediated Seed Coating to Enhance Germination

Seed coating represents a key strategy to enhance germination, stress tolerance, and early crop establishment. However, conventional formulations typically rely on synthetic polymers that raise environmental and safety concerns. Polyphenols have recently been explored as green and multifunctional alternatives, providing both protective barriers and responsive release capabilities (Table 2).

The integration of metal–phenolic coordination with MOFs has opened a route to smart antimicrobial seed coatings. For example, a UiO‐66 carrier loaded with the fungicide ipconazole is stabilized by a Zn^2+^–TA coordination shell, creating a dual‐responsive system capable of adapting to both pH and temperature fluctuations (Figure 8a). The Zn^2+^–TA shell acts as a protective coordination layer that prevents premature release and promotes stable adhesion on seed surfaces. Under slightly acidic conditions (e.g., pH ≈ 5–6), which mimic fungal infection, MPNs tend to disassemble, triggering the accelerated release of fungicides (Figure 8b). When applied to rice seeds, the Zn^2+^–TA‐coated formulation effectively suppressed *Fusarium fujikuroi* infection for up to 6 days across a wide range of pH, while maintaining germination rates above 90%. The Zn^2+^–TA layer not only strengthens structural integrity but also reduces leaching and nontarget microbial toxicity [112]. Core–shell MPN platforms provide a representative strategy for regulating the release of plant growth regulators under dynamic soil conditions through interfacial coordination. For example, solid lipid nanoparticles loaded with indole‐3‐acetic acid and coated with an Fe^3+^–TA shell exhibit pH‐ and salt‐responsive release behavior (Figure 8c) [133]. The network remains stable under neutral and alkaline conditions, but tends to disassemble under mild acidic or saline conditions, thereby aligning the release of indole‐3‐acetic acid with the physiological demand of germinating seeds. Similarly, wheat seeds coated with this formulation have shown enhanced germination rates and increased shoot activity under saline stress, thereby acting as both a biochemical modulator and a physical barrier (Figure 8d). Polyphenol‐derived nanostructures also provide an effective strategy for stress mitigation in seeds by integrating water regulation and redox buffering, while enabling physiological priming. For example, Ren et al. embedded TA‐derived carbon dots into biopolymer hydrogels to form polyphenol‐based seed coatings capable of alleviating drought stress during early germination. The hydrogel‐based coatings act as a transient moisture reservoir surrounding the seeds, while gradually releasing antioxidant‐active carbon dots that modulate ROS and enhance root water uptake (Figure 8e–h) [136].

**FIGURE 8:**
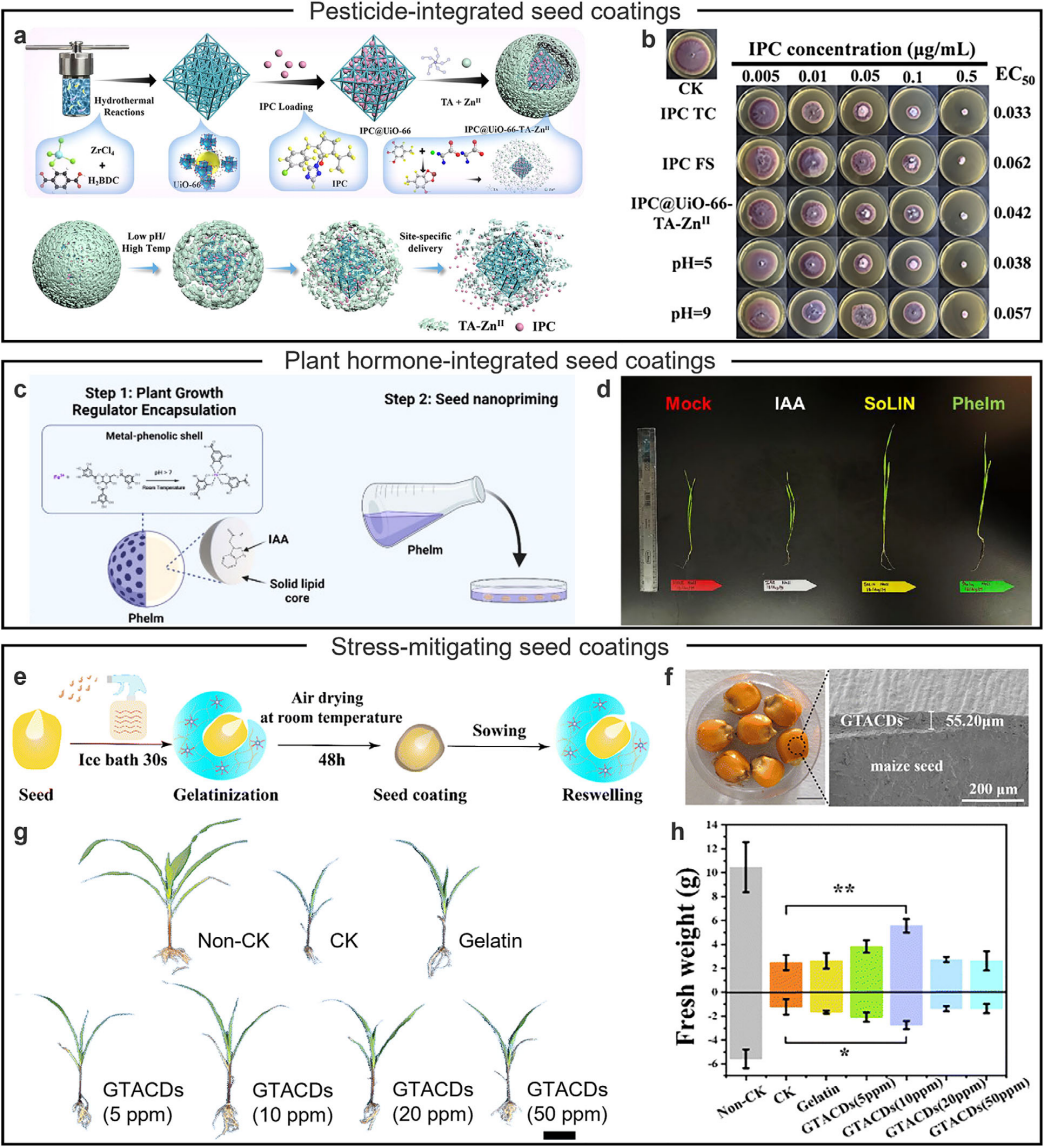
Polyphenol‐enabled materials as a platform for seed coating. (a) Schematic of the possible mechanism of ipconazole (IPC)@UiO‐66‐TA‐Zn^2+^ formation and schematic of IPC release from IPC@UiO‐66‐TA‐Zn^2+^ at various temperatures and pH values. (b) Fungicidal activities of IPC technical concentrate (TC), IPC@UiO‐66‐TA‐Zn^2+^, and IPC flowable suspension (FS) against *Fusarium fujikuroi* and IPC@UiO‐66‐TA‐Zn^2+^ against *Fusarium fujikuroi* under different pH conditions for 6 days at 25°C. EC_50_, 50% of mycelial inhibition. (a) and (b) Adapted with permission [112]. Copyright 2024 Zhang et al. (c) Preparation of the solid lipid interface (SoLIN) and phenolic network with a lipid core and metal‐coordinated shell for encapsulating indole‐3‐acetic acid (IAA). (d) Effect of nanopriming on wheat plants irrigated with saline stress (100 mM NaCl). The wheat plants were subjected to four treatments: a control (Mock), IAA, SoLIN, and Phelm (phenolic network with a lipid core and metal coordinated shell) nanopriming. (c) and (d) Adapted with permission [133]. Copyright 2024, American Chemical Society. (e) Schematic of seed coating and reswelling. (f) Photograph (scale bar 1 cm) and scanning electron microscopy image of seed coating. (g) Photographs of maize plants subjected to different treatments: non‐CK (non‐drought stress, soil moisture content 75%), CK (drought stress, soil moisture content 40%), gelatin, and TA‐derived carbon dot‐embedded gelatin hydrogels ((GTACDs) 5, 10, 20, 50 ppm). (h) Fresh weights of maize shoots and roots subjected to different treatments (non‐CK, CK, gelatin, GTACDs (5, 10, 20, 50 ppm)) after 30 days under drought stress. (e)–(h) Adapted with permission [136]. Copyright 2024, The Royal Society of Chemistry.

### 3.3 Polyphenol‐Mediated Nutrient Release

Nutrient management is central to sustainable agriculture. However, conventional fertilizers typically exhibit burst release of nutrients, leading to nutrient loss, low nutrient use efficiency, and environmental burdens. Although petroleum‐based CRFs have been developed to mitigate these issues, their poor degradability and dependence on nondegradable polymers raise concerns about secondary pollution and long‐term sustainability [173]. Coatings and composites assembled from polyphenols typically feature strong adhesion, environmental responsiveness, and biodegradability (Table 2). Therefore, polyphenol‐enabled materials can serve as a promising alternative to petroleum‐based CRFs, enabling controlled nutrient delivery.

Efficient nutrient delivery requires carriers that can form dense, stable coatings on highly soluble fertilizers, while maintaining tunable permeability. For example, during the preparation of controlled‐release urea, the introduction of moisture causes urea granules to dissolve, thereby affecting the final release performance. Therefore, developing a polyphenol‐based coating in a nonaqueous medium is required. We recently demonstrated the rapid self‐assembly of Fe^3+^–TA networks on urea granules in an organic solvent (i.e., acetonitrile) (Figure 9a), producing thick (2–25 µm) shells within minutes through a coordination‐driven condensation process (Figure 9b) [137]. The resulting coatings exhibit pH‐dependent disassembly of MPNs, providing controlled release of nitrogen, while releasing trace micronutrients (i.e., Fe^3+^) from the MPN coatings. The fluidized bed coating strategy has also been used to resolve the challenge of encapsulation when aqueous solutions cannot be used to form uniform coatings [174]. In this approach, solutions are atomized into fine droplets, which enables uniform deposition on particle surfaces. For example, using a fluidized bed process, oxidatively polymerized TA forms uniform poly(TA) shells that regulate urea release and improve nutrient use efficiency under pot conditions [142]. Similarly, sprayable formulations combining poly(vinyl alcohol) with Fe^3+^–TA networks can enhance membrane integrity and extend the nutrient‐release window to nearly 10 days, while preserving mechanical robustness in the field [140].

**FIGURE 9:**
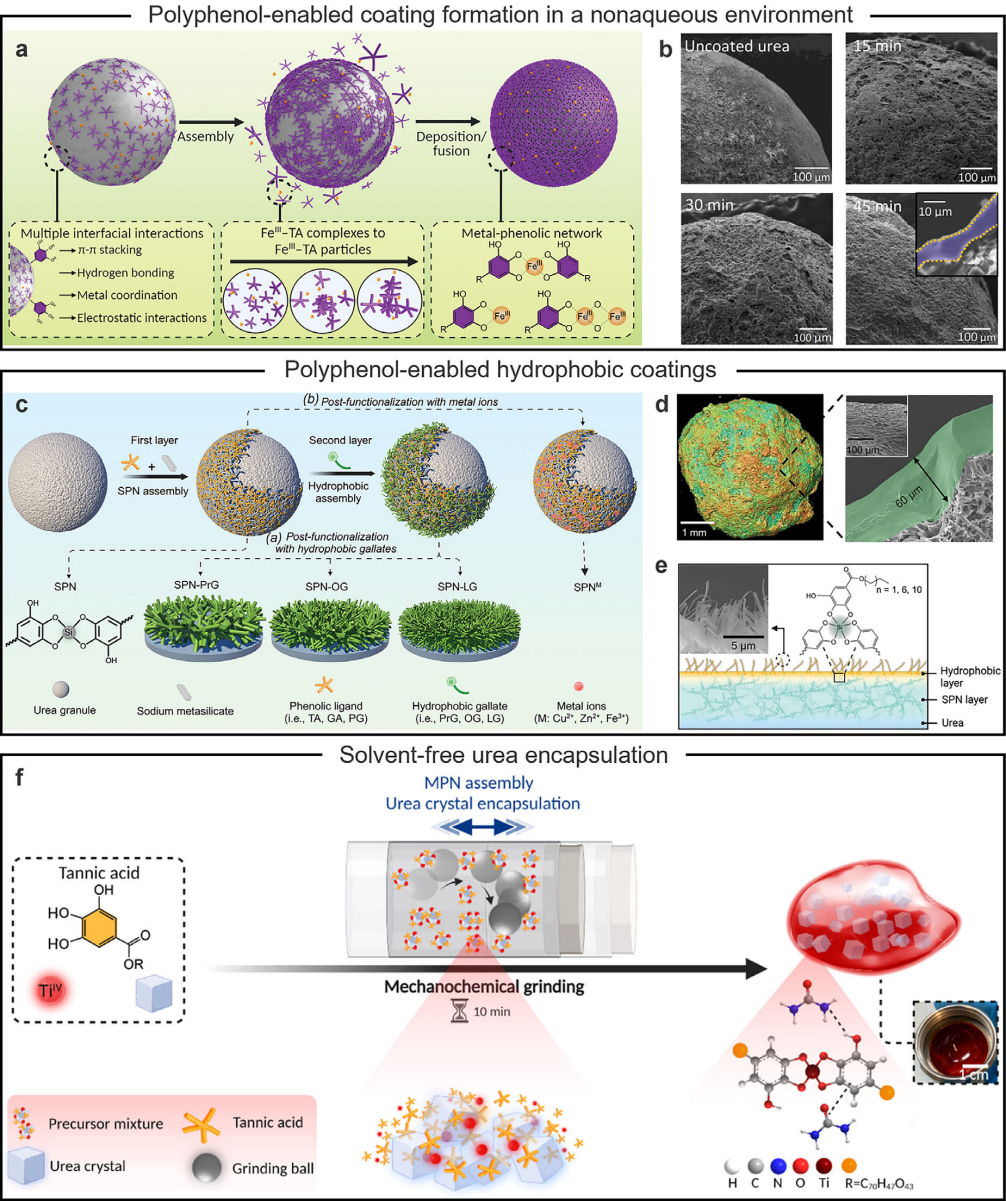
Nutrient delivery using polyphenol‐based materials. (a) Schematic of the MPN assembly protocol used to coat urea granules (water‐soluble substrate) in acetonitrile solution. (b) Scanning electron microscopy images of the surfaces of MPN‐coated urea granules showing the time‐dependent growth of the MPN film on urea granules following immersion of the urea granules in an Fe^3+^–TA solution. (a) and (b) Adapted with permission [137]. Copyright 2022 Mazaheri et al., published by Wiley‐VCH GmbH. (c) Schematic of the assembly of an SPN coating on a urea granule, and post‐functionalization of the SPN coating with hydrophobic gallates to engineer hydrophobic SPN coatings (SPN‐PrG, SPN‐OG, and SPN‐LG) or metal ions to engineer SPN^M^ coatings (where M is the metal ion). PrG, propyl gallate; OG, octyl gallate; LG, lauryl gallate, GA, gallic acid; PG, pyrogallol. (d) Micro‐computed tomography image of an SPN coating on a urea granule and false‐colored cross‐sectional image. (e) Schematic of the interfacial interactions between the SPN layer and the hydrophobic layer. (c)–(e) Adapted with permission [115]. Copyright 2024, Wiley‐VCH GmbH. (f) Schematic of the mechanochemical coordination‐driven assembly of MPNs for encapsulating urea crystals. Adapted with permission [176]. Copyright 2023, American Chemical Society.

Polyphenol‐based coatings typically exhibit high hydrophilicity, which influences the performance of CRFs. To address this, SPNs provide a programmable coating strategy to regulate water transport and nutrient release more precisely. We introduced dynamic SPN coatings through condensation between sodium metasilicate (Na_2_SiO_3_) and polyphenols (Figure 9c–e) and employed hydrophobic polyphenol ligands (i.e., propyl gallate, octyl gallate, and lauryl gallate) to modify the SPN coatings. The resulting SPNs exhibited two distinct pH‐response windows (pH < 5 and pH > 10). Urea was released within 7–14 days, depending on coating hydrophobicity, while silicon persisted up to 28 days in soil. The Cu^2+^‐, Fe^3+^‐, Al^3+^‐, and Ti^4+^‐doped SPNs showed a staged release pattern over approximately 2 weeks. This SPN platform establishes a chemically programmable framework in which macro‐ and micronutrients follow decoupled diffusion pathways governed by coordination strength and dynamic bonding [115]. Polymers (e.g., poly(dimethylsiloxane)) have also been combined with polyphenols to develop stronger, more hydrophobic coatings for encapsulating CRFs [139]. For example, Wang et al. have developed multi‐trigger systems such as Fe^3+^–TA‐modified polyacrylate coatings that integrate pH‐responsive coordination and improve resistance to nutrient leaching [175]. Introducing long‐chain alkyl groups is a common method for hydrophobic modification to enhance the hydrophobicity of coatings. Long‐chain alkyl groups are introduced into the polyphenols via Michael or Schiff base reaction [141]. Although this approach enhances hydrophobicity, it reduces the mechanical strength of the coatings [143].

Solvent‐free encapsulation represents an emerging strategy for nutrient delivery using polyphenol‐based materials, as it avoids solvent residues while enabling high loading efficiency and structural robustness. We have shown that MPNs can be directly assembled in the solid state to encapsulate urea, with coordination between polyphenols and metal ions proceeding under mechanical activation without the need for solvents. This solid‐state process yields dense and uniform MPN shells that effectively encapsulate urea, suppress premature dissolution, and enable sustained nutrient release under aqueous conditions. Beyond improving encapsulation efficiency, solvent‐free MPN engineering provides a scalable and environmentally benign route for controlled nutrient delivery, aligning polyphenol chemistry with the practical constraints of fertilizer manufacturing and field application (Figure 9f) [176].

Quantitative release modeling remains underdeveloped in polyphenol‐based fertilizer systems, where cumulative release profiles are typically used. For alkylamine–poly(TA)‐coated urea, the Shaviv diffusion model was used to quantify nitrogen release by incorporating the nitrogen and water‐vapor permeability coefficients of different alkylamine‐modified coatings. The model predicted 60% nitrogen release after 63, 40, 24, and 17 days for the octadecylamine‐, hexadecylamine‐, dodecylamine‐, and hexylamine‐modified coatings, respectively; the predicted release times were comparale to the corresponding experimental release times of 55, 48, 20, and 15 days [141]. In biodegradable Fe^3+^–TA/carboxymethyl cellulose bioplastics fertilizers, Fe^3+^ release was fitted with pseudo‐second‐order, Higuchi, and Korsmeyer–Peppas models, with the pseudo‐second‐order model giving the best fit (*R* ^2^ = 0.99; kinetic constant (*K*) = 0.52 mg g^−1^ h^−1^) [177].

### 3.4 Polyphenol‐Mediated Pesticide Delivery for Plant Protection

Pesticides are essential for enhancing crop yield and ensuring food security. However, conventional formulations are typically limited to rapid photodegradation, leaching, and low utilization, resulting in environmental risks and efficiency reduction. The abundant catechol and galloyl moieties in polyphenol‐based platforms provide versatile interaction motifs for pesticide encapsulation and controlled release (Table 2), while simultaneously incorporating supporting functions such as interfacial adhesion, photoprotection, and catalytic activity [144]. These intrinsic properties make polyphenol‐mediated delivery strategies compatible with pesticides featuring distinct molecular structures and application pathways. Polyphenols can function either as carriers to enhance loading efficiency, stabilization, and surface retention, or as surface coatings that improve solid‐state encapsulation and regulate diffusion‐dominated release. When combined with different application protocols, including root‐targeted delivery and foliar application, polyphenol‐based systems enable localized efficacy, reduced off‐target loss, and improved rain fastness compared to conventional spray coatings, while also exposing limitations, including short‐term release, environmental risks, and instability in complex soil environments (Figure 10).

**FIGURE 10:**
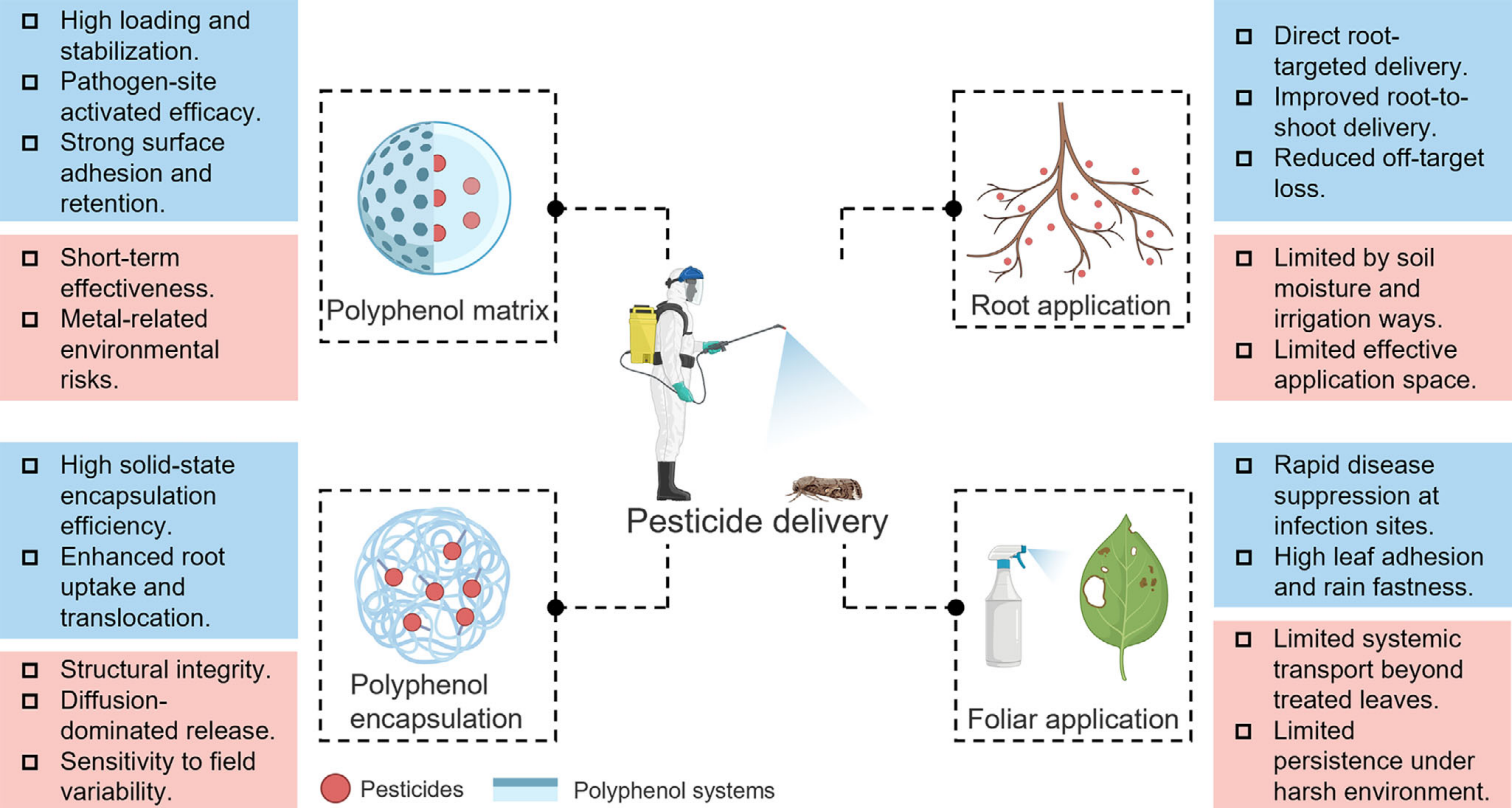
Polyphenol‐mediated pesticide delivery in agricultural systems. The main technologies for the delivery of pesticides based on polyphenols, including matrix and encapsulation are highlighted along with the different pathways for the utilization of polyphenol‐based pesticides, including root and foliar applications. Created in BioRender.com. Liu, H. (2026) https://BioRender.com/ya6gd7l.

Polyphenol assemblies as a matrix regulate pesticide release through catalytic activation or environment‐triggered responsiveness. For instance, metal–TA complexes have been engineered into CuO_2_ nanoclusters that act as self‐supplying ROS nanogenerators (Figure 11a). These assemblies catalytically convert endogenous H_2_O_2_ to •O_2_^−^, generating oxidative stress that inhibits pathogens (e.g., *Pseudomonas syringae* and *Fusarium oxysporum*) (Figure 11b) [145]. Similarly, TA can etch and functionalize zeolitic imidazolate frameworks (e.g., ZIF‐8), yielding hollow ZIF‐8@TA hybrids with high boscalid loading and pH‐responsive release in the acidic environment of *Botrytis cinerea*, which simultaneously enhances foliar adhesion and antifungal efficacy (Figure 11c) [178]. Kinetic fitting showed that boscalid release was best described by the Ritger–Peppas model across pH 5.5–8.5 and release exponent values below 0.43, indicating Fickian diffusion‐controlled release from the hollow ZIF‐8@TA matrix [178]. In addition, by further introducing a second transition metal into the ZIFs, pore structure and defect density can be modulated, as shown by bimetallic ZnM‐ZIFs (M = Cu, Ni) coated with Fe^3+^–TA networks. The resulting ZnM‐ZIF@MPN carriers show photostability and controllable pH‐responsive release [150]. Moreover, azadirachtin–TA co‐assembled nanoparticles were fitted using first‐order, Ritger–Peppas, Higuchi, and Peppas–Sahlin models. The Higuchi model provided the best fit, with *R* ^2^ values of 0.879–0.995, suggesting dissolution‐ and diffusion‐associated release behavior, while Ritger–Peppas exponents below 0.45 further indicated Fickian diffusion [179]. In another study, metal–phenolic‐coated rod‐like silica nanocarriers were reported to show pH‐responsive pesticide release, reaching approximately 70% release after 72 h of stimulation, with the release process described as first‐order kinetics [180].

**FIGURE 11:**
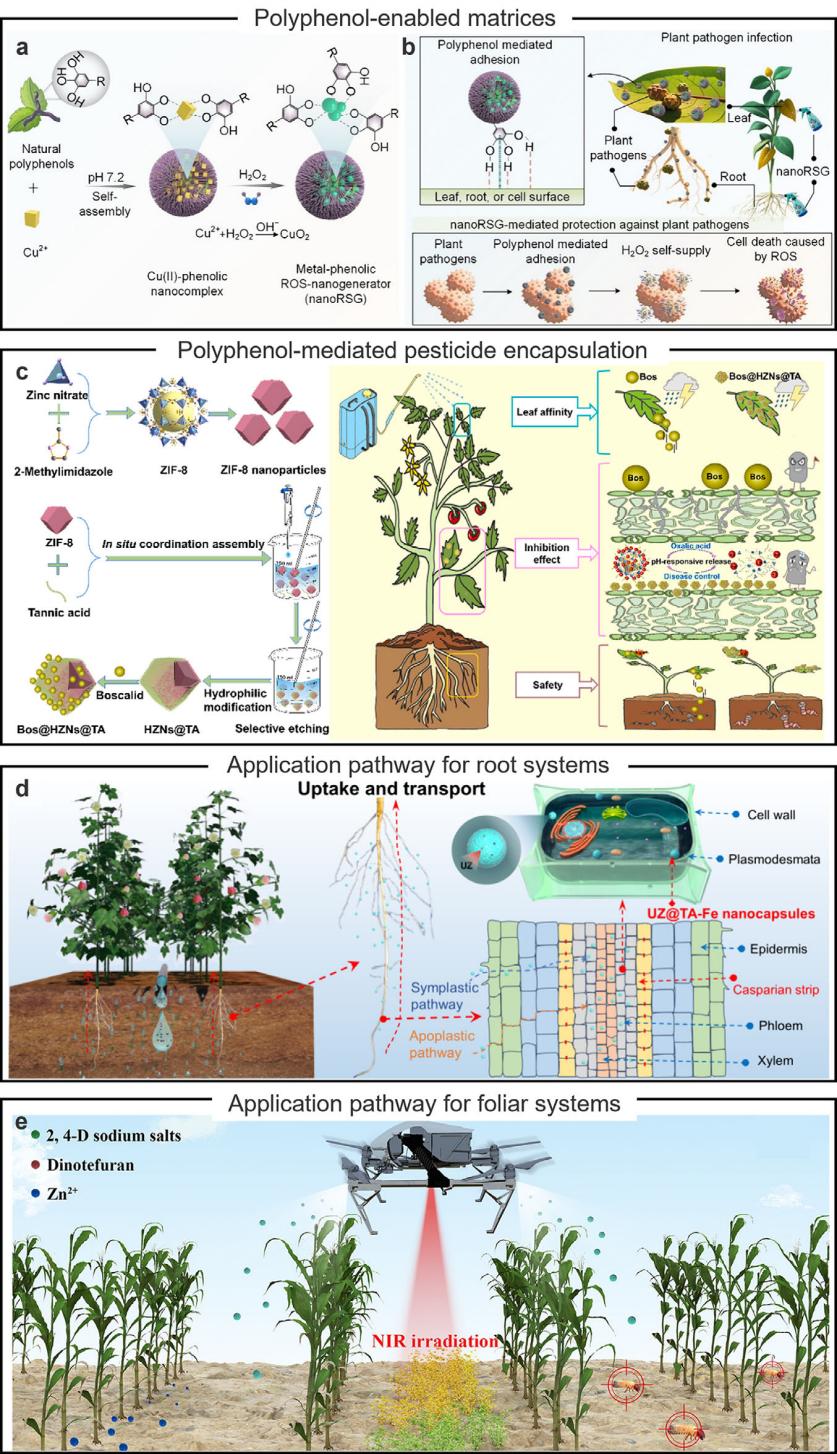
Pesticide delivery realized from polyphenol‐based materials. (a) Schematic of the synthesis of a metal–phenolic ROS–nanogenerator (nanoRSG). (b) nanoRSG‐mediated protection against plant pathogens. (a) and (b) Adapted with permission [145]. Copyright 2025, American Chemical Society. (c) Schematic preparation of boscalid (Bos)‐encapsulated hollow zeolitic imidazolate framework‐8 nanoparticles (HZNs) and TA hybrids (Bos@HZNs@TA) and their application for pesticide delivery in tomato plant cultivation. Adapted with permission [178]. Copyright 2023, Elsevier Ltd. (d) Uptake and transport diagram of uniconazole@TA‐Fe (UZ@TA‐Fe) nanocapsules in cotton roots. Adapted with permission [144]. Copyright 2025, American Chemical Society. (e) Scheme of the application of hollow mesoporous silica@PDA@zeolitic imidazolate framework and its synergistic effects of weeding, insecticide, and nutrient supply. Adapted with permission [183]. Copyright 2021, American Chemical Society.

The release behavior of polyphenol‐based pesticides is also governed by local physicochemical properties at soil–root and leaf–air interfaces. For root‐applied pesticide delivery, mechanochemically prepared uniconazole@TA–Fe nanocapsules provided a quantitative example of structure‐regulated release from a metal–polyphenol shell (Figure 11d) [144]. The optimized uniconazole@TA–Fe(zirconia) nanocapsules exhibited a porous core–shell structure with a high uniconazole loading (89.3%), an average particle size of 467 nm, and an average pore diameter of 38 nm. Kinetic modelling showed that uniconazole release followed first‐order kinetics and was governed by dissolution and diffusion, while imaging analyses confirmed the internalization of the nanocapsules by cotton roots, including their entry into root‐tip and main‐root cells [144]. In foliar applications, conventional pesticide delivery technologies are limited by low utilization efficiency due to poor adhesion, UV radiation, and quick diffusion after rainfall or irrigation. Recent advances based on polyphenols have addressed these challenges through polyphenol–metal coordination and the establishment of a hydrophobic interface. For example, Fe^3+^–TA‐gated MOF nanopesticides respond to pH, H_2_O_2_, and glutathione while maintaining excellent dispersibility and degradability [181]. The integration of MOFs with MPNs further combines the high porosity and structural tunability of MOFs with the adhesion of MPNs [182]. MPN‐coated MOFs exhibit improved wettability and adhesion through hydrogen bonding and metal coordination, thereby prolonging residence time under rainfall [149]. Polyphenol‐based materials provide a versatile platform for environmentally responsive release. For example, Ji et al. proposed an all‐in‐one nanoplatform that integrates pesticide encapsulation, adhesion improvement, and stimuli‐responsive release, while employing deep‐learning algorithms to optimize structural parameters to enhance foliar retention (Figure 11e) [183]. Rice seed coating formulations, such as Zn^2+^–TA nanocomposites, further demonstrate how metal–phenolic engineering can be extended to the plant interface for precise pathogen management. Specifically, the Zn^2+^–TA coating acts as a dynamic trigger that regulates fungicide release in response to environmental stimuli such as pH and temperature, while simultaneously enhancing adhesion to seed surfaces and facilitating nanoparticle penetration into both plant tissues and fungal cells, thereby improving antifungal efficacy and reducing off‐target toxicity [112]. Similarly, Fe^3+^–TA nanocapsules integrate ROS, enzymatic, and photothermal responsiveness, achieving the precise release of fungicides under multiple environmental triggers [147].

Polyphenol‐based systems have intrinsic antimicrobial activity arising from redox chemistry and metal‐mediated ROS generation. While this redox activity can benefit pathogen inhibition, uncontrolled ROS production may cause oxidative damage to redox‐sensitive cargos and plant tissues. As this redox behavior is strongly dependent on polyphenol structure, polyphenol selection can be used to balance antioxidant–pro‐oxidant effects. For example, Moilanen and Salminen systematically compared 27 plant ellagitannins and showed that the in vitro oxidative activity of such polyphenols at high pH depends on specific structural features, including valoneoyl groups, acyclic glycosyl substituents, nonahydroxytriphenoyl groups, sanguisorboyl groups, and hexahydroxydiphenoyl groups [184]. Ellagitannins have further been shown to exhibit antioxidant, pro‐oxidant, and metal‐chelating activities [185]. Catechol and galloyl groups coordinate with transition metals (e.g., Cu^2+^) to initiate Fenton‐like reactions that generate hydroxyl radicals and induce oxidative membrane damage [145]. The mechanistic foundation of this phenomenon has been clarified by Xu et al., who propose that catechol and galloyl groups reversibly oxidize to quinones while forming dynamic coordination complexes with Fe^3+^ or Cu^2+^. This framework explains how the chemical reactivity of polyphenols contributes to the antibacterial activity observed in chemodynamic nanopesticides [99]. Plant‐derived polyphenols (e.g., resveratrol and coumarin) also demonstrate comparable bactericidal effects against *Ralstonia solanacearum*, suggesting that naturally occurring polyphenols can provide functions similar to those of synthetic polyphenol‐based systems [186]. Upon coordination with metal ions to form MPNs, this antioxidant–pro‐oxidant balance can be further modulated by varying metal ions. Redox‐active metal ions, such as Fe^3+^ and Cu^2+^, can promote localized ROS generation via redox cycling and are therefore beneficial for antimicrobial applications [187]. In contrast, redox‐inert or low‐Fenton‐activity metal ions (e.g., Zn^2+^) are favored for applications involving redox‐sensitive cargos or living microbial inoculants, where minimizing ROS generation is essential for preserving cargo integrity and viability. Collectively, the combined antioxidant and pro‐oxidant properties of polyphenols, along with their metal‐chelating capability, highlight that the antioxidant properties of polyphenols should not be regarded in isolation in materials design. Instead, their overall redox behavior arises from the interplay between polyphenol chemistry, incorporated guest species (e.g., metal ions and cargo), synthesis conditions (e.g., pH, temperature, and oxygen availability), and the intended application environment. For agricultural applications in particular, consideration should be given to soil conditions, plant compatibility and the desired level of ROS generation so that polyphenol‐enabled materials can promote beneficial redox signaling while minimizing oxidative damage.

### 3.5 Polyphenol‐Mediated Sensing and Monitoring

Rapid and reliable detection of agrochemicals and food contaminants is essential for sustainable agriculture and food safety. Conventional analytical methods usually rely on sophisticated instruments and complicated procedures, limiting their applicability for on‐site monitoring [188]. Polyphenols, owing to their near‐universal binding affinity and high biocompatibility, have emerged as versatile building blocks for engineering green sensors. They provide multiple functions ranging from signal transduction and catalytic amplification to structural stabilization, enabling sensitive, selective, and sustainable sensing technologies (Table 2).

Traditional colorimetric sensors rely on pH‐responsive dyes or single‐stage reactions, which often suffer from poor stability and limited color range. Inspired by the reversible coordination and redox chemistry of polyphenols, Cheng et al. have developed a metal–polyphenol multistage competitive coordination system that translates subtle chemical changes into distinct optical responses (Figure 12a). The polyphenols dynamically coordinate to multiple metal ions and volatile amines, producing sequential color transitions that reflect the degree of food spoilage. This approach achieves rapid, noninvasive, and remote freshness assessment of meat products via smartphone‐based imaging, representing a convergence of chemical sensing and digital intelligence (Figure 12b) [152]. Another study has used the tunable optical properties of metal–polyphenol assemblies for environmental monitoring. Cu^2+^–polyphenol nanosheets were engineered with peroxidase‐like and photothermal activity, enabling sensitive colorimetric detection of hydrogen peroxide and related ROS [154].

**FIGURE 12:**
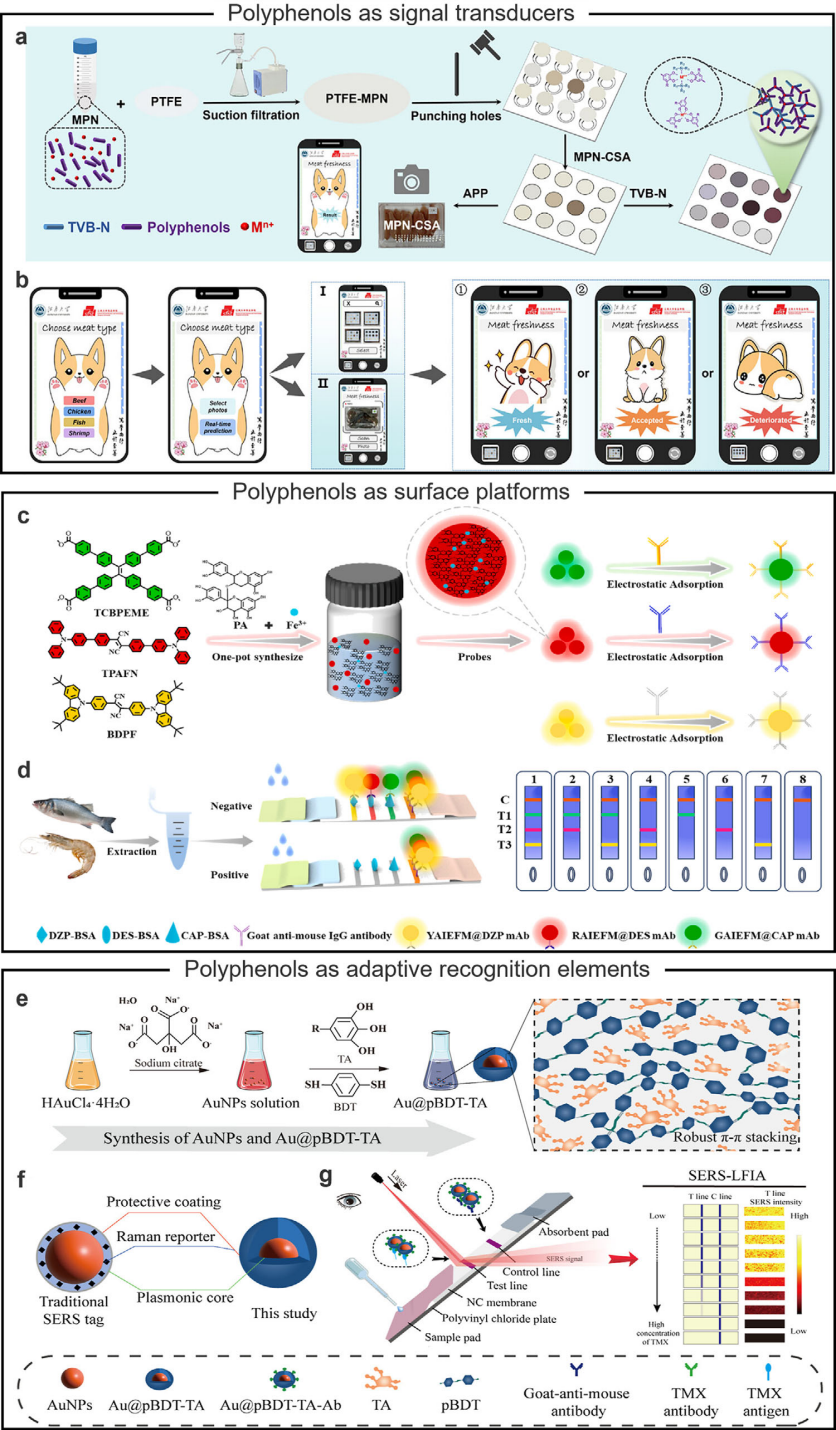
Role of polyphenols in agricultural biosensing. (a) Schematic of the preparation of an MPN colorimetric sensor array (MPN‐CSA) and establishment of a smart monitoring platform. PTFE, polytetrafluoroethylene; TVB‐N, total volatile basic nitrogen; APP, application. (b) Application of the detection platform for the accurate monitoring of meat food freshness by both remote image recognition and real‐time online scanning. (a) and (b) Adapted with permission [152]. Copyright 2025, Wiley‐VCH GmbH. (c) Schematic of the multiplex aggregation‐induced emission fluorescent microspheres (AIEFMs)‐immunochromatographic assay (ICA) for the detection of chloramphenicol (CAP), diethylstilbestrol (DES), and diazepam (DZP). TCBPEME, tetramethyl 4′,4‴,4‴″,4‴⁗‐(ethene‐1,1,2,2‐tetrayl)‐tetrakis([1,1′‐biphenyl]‐4‐carbomethoxy); TPAFN, 2,3‐bis(4′‐(diphenylamino)‐[1,1′‐biphenyl]‐4‐yl)fumaronitrile; BDPF, 2,3‐bis(4‐(3,6‐ditert‐butyl‐9H‐carbazol‐9‐yl)‐phenyl)fumaronitrile; PA, proanthocyanidin. (d) Detection principle of the multiplex AIEFMs‐ICA for simultaneous detection of CAP, DES, and DZP. (c) and (d) Adapted with permission [153]. Copyright 2025, Elsevier B.V. (e) Synthesis of Au@pBDT‐TA and an enlarged view of the coating for pBDT‐TA. pBDT, polymerized benzene‐1,4‐dithiol. (f) Comparison between a traditional surface‐enhanced Raman scattering (SERS) tag and the polyphenol‐mediated SERS tag. (g) Principle of Au@pBDT‐TA‐based SERS–lateral flow immunoassay (LFIA) for the detection of thiamethoxam (TMX). (e)–(g) Adapted with permission [156]. Copyright 2024, American Chemical Society.

In pesticide and veterinary drug detection, polyphenol‐assisted micro‐ and nanostructures enable improved fluorescence and bio‐interface stability. For instance, Su et al. constructed a multiplex immunochromatographic assay using tricolor aggregation‐induced emission fluorescent microspheres encapsulated within polyphenol–Fe^3+^ networks. Three detection channels (i.e., green, yellow, and red) enabled simultaneous quantitative analysis of chloramphenicol, diethylstilbestrol, and diazepam on a single test strip (Figure 12c). The MPN coating enhanced photostability and antibody immobilization efficiency without additional coupling agents, affording pg mL^−1^‐level sensitivity and accurate on‐site analysis of aquatic products (Figure 12d) [153]. Complementary strategies have leveraged polyphenol‐modified solid‐phase or electrochemical platforms for pesticide residue analysis. For instance, TA‐etched Fe@ZIF‐8 composites were used for magnetic solid‐phase extraction coupled with high‐performance liquid chromatography, providing a green and efficient method to quantify benzoylurea insecticides in fruit juices [189]. Furthermore, polyphenol‐based nanozymes offer catalytic amplification, as demonstrated by lignin‐derived Fe single‐atom nanozymes combined with acetylcholinesterase, which enabled colorimetric sensing of organophosphorus pesticides in soil [157].

Polyphenols have also been exploited in plasmonic amplification platforms to achieve ultrasensitive detection of small‐molecule contaminants. For example, Chen et al. reported a surface‐enhanced Raman scattering–lateral flow immunoassay for the rapid detection of thiamethoxam in tea, employing a Au@polymerized benzene‐1,4‐dithiol (pBDT)‐TA nanostructured tag (Figure 12e–g). The polyphenol‐derived pBDT–TA coating formed a uniform and conductive shell on gold nanoparticles, which resulted in enhanced local surface‐plasmon resonance and improved signal reproducibility (Figure 12e,f). Compared to conventional surface‐enhanced Raman scattering tags, this architecture afforded a tenfold increase in Raman intensity and a detection limit of 0.05 ng mL^−1^, while maintaining high selectivity against structurally similar neonicotinoids (Figure 12g) [156]. Recent studies have further expanded their sensing role toward sustainable electrode platforms coupled with biological recognition elements. Lignin‐based screen‐printed electrodes functionalized with microalgae have been proposed for photoelectrochemical sensing of sustainable nano‐herbicides, integrating biological recognition with eco‐designed polyphenol supports [190].

### 3.6 Polyphenol‐Enabled Nitrification Inhibition

Nitrogen losses through ammonia volatilization, nitrification, nitrate leaching, and denitrification account for more than half of the applied nitrogen loss into the environment, causing both agronomic inefficiency and ecological degradation [1]. Synthetic urease and nitrification inhibitors (e.g., *N*‐(*n*‐butyl)thiophosphoric triamide (NBPT) and 3,4‐dimethylpyrazole phosphate) have been widely adopted to mitigate nitrogen losses [191]. However, their application is limited by high costs and environmental risks. Polyphenols offer a sustainable alternative to synthetic inhibitors (Table 2). Their ability to chelate transition metals and interact with microbial enzymes allows them to mimic biological nitrification inhibition (BNI), while enabling molecular and material‐level regulation of nitrogen transformation in soil. The concept of BNI offers a mechanistic foundation for designing polyphenol‐based inhibitors (Figure 13a) [192].

**FIGURE 13:**
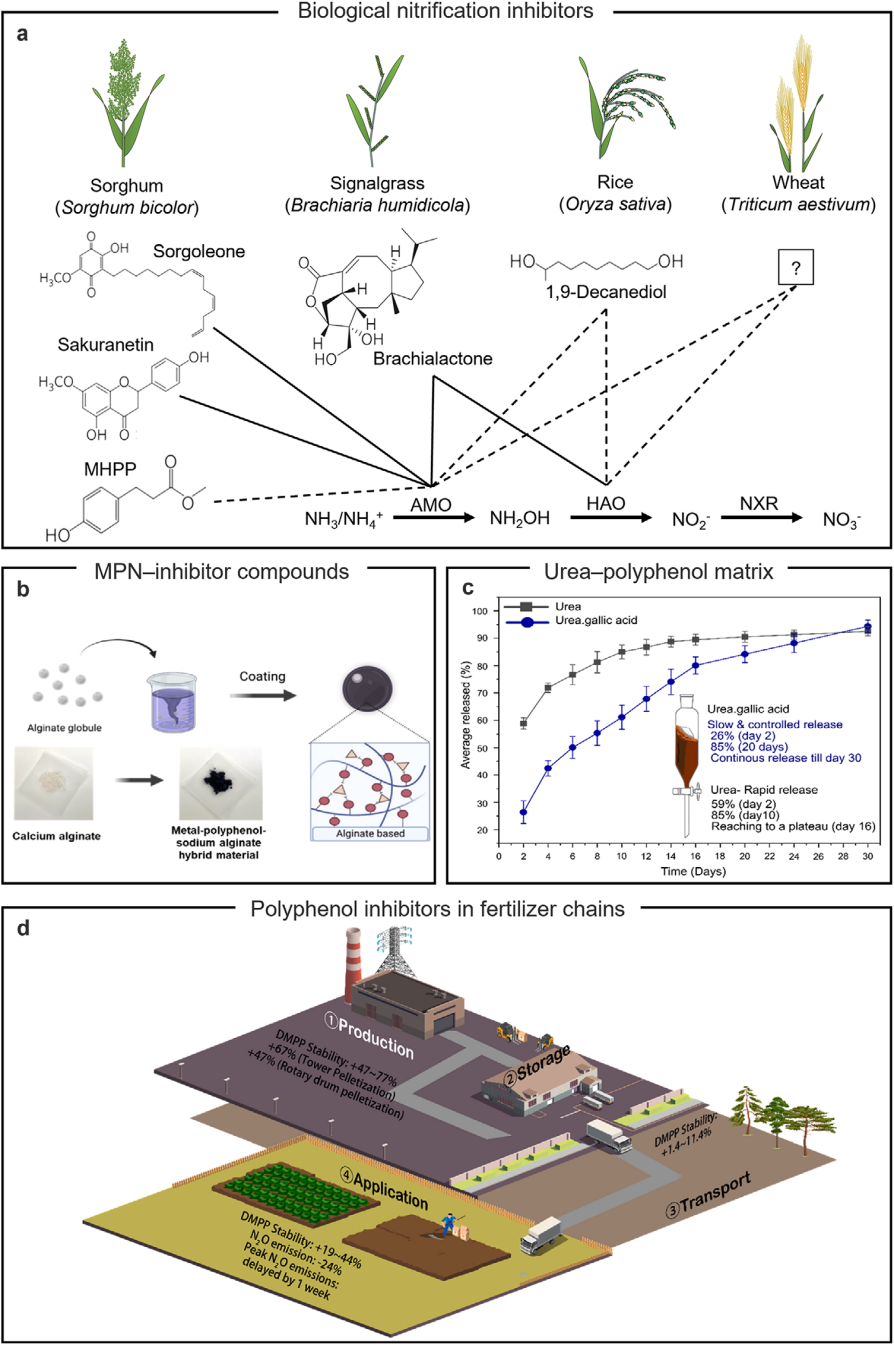
Examples of polyphenol‐based materials as nitrogen inhibitors. (a) BNIs from root exudates and their enzyme targets. Adapted with permission [192]. Copyright 2017, Macmillan Publishers Limited. (b) Preparation of alginate–metal–polyphenol nanoparticles. Adapted with permission [158]. Copyright 2024, Elsevier Ltd. (c) Cumulative urea release profiles of commercial urea and urea–gallic acid (molar ratio of urea and gallic acid is 1:1) (*n* = 3). Adapted with permission [160]. Copyright 2025, American Chemical Society. (d) Schematic of the alginate–metal–polyphenol system on the stability of 3,4‐dimethylpyrazole phosphate (DMPP) in the enhanced‐efficiency fertilizers chain. Adapted with permission [158]. Copyright 2024, Elsevier Ltd.

Root exudates of plants (e.g., *Brachiaria* and *Sorghum*) have been shown to naturally suppress the activity of *Nitrosomonas* and *Nitrobacter* by targeting ammonia monooxygenase and hydroxylamine oxidoreductase, key enzymes that are responsible for the oxidation of NH_4_^+^ to NO_2_^−^ (Figure 13a). The active BNI compounds typically contain phenolic groups or quinonoid structures that can interact with nitrification enzymes, such as ammonia monooxygenase, potentially disrupting electron transfer processes and inhibiting ammonia oxidation [192]. These chemical features parallel the structural motifs of natural polyphenols (e.g., catechols, tannins, and flavonoids), which possess hydroxyl and carbonyl groups that can coordinate transition metals and interfere with urease or oxidase activity. Field and incubation studies have also indicated that polyphenols from tea exhibit a stronger inhibition on nitrification than denitrification, mainly by reducing the abundance of ammonia‐oxidizing archaea and bacteria, confirming their potential for targeted nitrification control [193]. Structure–activity analyses have further identified hydroxyl group orientation on flavanols, scutellarin, and quercetin analogues as a key driver for enzyme binding efficiency [194, 195]. Recent studies have revealed that chlorogenic, caffeic, and luteolytic acids derived from dandelion serve as effective inhibitors [196], while tannin‐rich barks (e.g., *Stryphnodendron adstringens*) show inhibition comparable to NBPT [197].

Diverse polyphenol‐enabled platforms are increasingly explored to enhance nitrogen use efficiency by stabilizing fertilizers and mitigating nitrogen loss in soil. For example, Li et al. constructed alginate–metal–polyphenol microenvironments to encapsulate 3,4‐dimethylpyrazole phosphate, which improved its thermal stability by 47%–77% at 70°C–125°C and reduced N_2_O emissions by 26% under field conditions (Figure 13b) [158]. Importantly, this stabilization has been shown to persist throughout the fertilizer value chain, from industrial production and storage to transport and field application, demonstrating that polyphenol‐enabled microenvironments can preserve inhibitor functionality beyond laboratory conditions (Figure 13d). This alginate–metal–polyphenol strategy highlights a scalable route for translating nitrification inhibitors into industrially compatible, low‐risk fertilizer products. Another study on crystal engineering has extended polyphenol inhibitors, building on earlier findings that catechols can irreversibly deactivate urease through covalent binding to cysteine residues [198]. Most recent progress has involved the formation of urea–polyphenol cocrystals, which combine nitrogen supply with intrinsic inhibition capacity. The urea–gallic acid cocrystal developed by mechanochemical synthesis represents a benchmark system. The incorporation of gallic acid alters the hydrogen‐bonding network of urea, resulting in reduced solubility and moderated hydrolysis rates (Figure 13c). This urea–gallic acid cocrystal simultaneously reduces NH_3_ volatilization and maintains steady nutrient release to plants. Soil incubation and pot experiments have revealed that urea–gallic acid reduces cumulative nitrogen loss by more than twice than with urea, while promoting root elongation and chlorophyll accumulation [160].

The use of polyphenols as nitrification inhibitors has attracted growing interest; however, their persistence and mode of action under real soil conditions remain unclear. We note that although inhibitory effects can be observed in controlled assays, polyphenols may show inconsistent performance in fields because they can be rapidly transformed through microbial degradation, oxidation, adsorption onto minerals and organic matter, and complexation with metal ions, all of which reduce their bioavailable fraction. As a result, it remains unclear whether polyphenols directly inhibit nitrifying microorganisms or act indirectly by modifying soil physicochemical conditions, such as oxygen diffusion, redox balance, metal availability, or enzyme activity.

### 3.7 Polyphenol‐Mediated Food Preservation and Shelf‐Life Extension

Food spoilage caused by oxidation and microbial contamination is a significant challenge in storage and supply chains, resulting in nutrient loss and economic waste. Conventional preservatives and synthetic films (e.g., polyethylene, polypropylene, and polyvinyl chloride) may cause health and environmental concerns, highlighting the need for safe, biodegradable, and multifunctional alternatives. Polyphenols act as powerful antioxidants and antimicrobials. Recent studies have shown their various applications in hydrogels, nanoparticles, and nanocrystals for food preservation, where polyphenols provide dual protection and enhanced stability (Table 2).

Hydrogels are 3D hydrophilic cross‐linked networks that exhibit high water‐retention capacity and effective inhibition of microbial growth on food surfaces. A representative hydrogel constructed from quercetin, oxidized polysaccharides, and Zn^2+^ forms a cohesive and sprayable network through dynamic coordination (Figure 14a). The hydrogel establishes a protective layer on fruit surfaces, showing growth inhibition of *Escherichia coli* and *Staphylococcus aureus* (Figure 14b). When applied to blueberries, the coating effectively retains surface gloss and reduces microbial colonization throughout refrigerated storage (Figure 14c) [161]. The polyphenol‐based platform integrates antioxidant, antibacterial, and moisture‐retention capabilities within a biodegradable matrix, providing a sustainable alternative to petroleum‐based films. Beyond hydrogel‐based systems, polyphenol‐based nanoparticles provide another material platform for constructing antioxidant and antimicrobial barriers on fruit surfaces. For example, tea polyphenols self‐assembled with proteins and metal ions via hydrogen bonding and π–π interactions enable the formation of uniform, transparent nanocomposite layers on fruit surfaces (Figure 14d). The tea polyphenols and zinc ions nanocomposite coating exhibit strong radical‐scavenging capacity and broad‐spectrum antibacterial activity, while also enhancing moisture retention and gas‐barrier performance (Figure 14e). In storage tests, coated strawberries and bananas retain a higher vitamin C content and display reduced weight loss compared with uncoated controls, confirming that the self‐assembled film effectively delays both oxidative and microbial spoilage [84]. A further nanoscale material platform is represented by polyphenol‐based nanocrystals, which integrate metal–phenolic coordination with crystalline nanostructures to enable multifunctional food preservation. MPN‐modified chitin nanocrystals reinforce biomass packaging films, while imparting antioxidant activity, visual monitoring capability, and photothermal‐assisted antimicrobial effects, transforming passive materials into active and responsive preservation systems (Figure 14f,g) [166].

**FIGURE 14:**
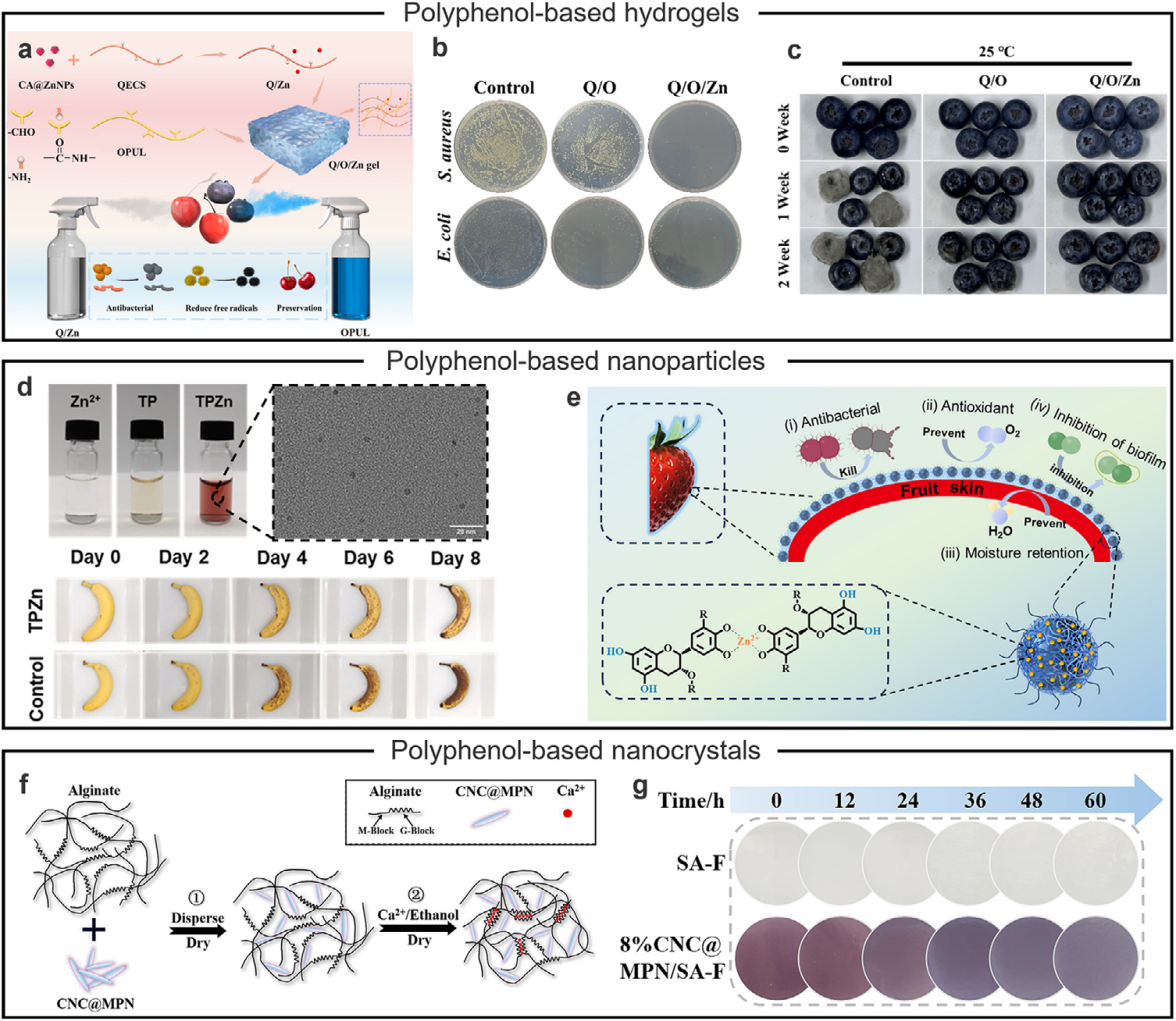
Examples of polyphenol‐based materials for food preservation and extension of shelf‐life. (a) Preparation of a polysaccharide hydrogel (Q/O/Zn hydrogel constructed from quercetin, oxidized polysaccharide, and Zn^2+^) loaded with chlorogenic acid (CA)–Zn^2+^ nanoparticles ((CA@ZnNPs)) and schematic showing the application of the hydrogel in fruit preservation. QECS, quaternized insect chitosan; OPUL, oxidized pullulan. (b) Optical images of colonies after coculture of the hydrogel with *Staphylococcus aureus* (*S. aureus*) and *Escherichia coli* (*E. coli*). (c) Evaluation of the freshness preservation activity of blueberries by the hydrogel. (a)–(c) Adapted with permission [161]. Copyright 2024, Elsevier Ltd. (d) Preparation and (e) preservation mechanism of tea polyphenols and zinc ions (TPZn) nanocomposite coating for fruit preservation. (d) and (e) Adapted with permission [84]. Copyright 2025, American Chemical Society. (f) Schematic of the preparation of metal–polyphenol network‐modified chitin nanocrystal films (CNC@MPN). (g) Digital photographs of sodium alginate films (SA‐F) and 8% metal–polyphenol network‐coated chitin nanocrystal/sodium alginate composite films (CNC@MPN/SA‐F) submerged in milk as a function of storage time. (f) and (g) Adapted with permission [166]. Copyright 2024, Elsevier B.V.

Oxidative degradation remains a primary driver of food deterioration, and polyphenols act as effective antioxidants through radical scavenging and metal chelation. Their incorporation into biopolymer matrices enhances oxidative resistance and extends product shelf life. For example, chitosan films grafted with gallic acid exhibit higher antioxidant capacity and tensile strength than chitosan films [199], while caffeic acid–genipin–chitosan films maintain radical‐scavenging activity under acidic conditions [200]. Zein–chitosan composite films containing phenolic acids exhibit enhanced 2,2‐diphenyl‐1‐picrylhydrazyl and 2,2′‐azino‐bis(3‐ethylbenzothiazoline‐6‐sulfonic acid) radical‐scavenging capacities compared with zein films, thereby improving oxidative stability during long‐term storage [201]. Beyond their antioxidation and antimicrobial activities, polyphenols play an essential role in enhancing the mechanical robustness of food packaging through covalent grafting and metal–phenolic coordination. Self‐assembled MPNs have been employed as additional layers in biopolymer films, which concurrently improve tensile strength, barrier performance, and biological functionality [165]. For example, carrageenan packaging films reinforced by MPNs exhibit enhanced tensile strength, barrier properties, and bioactivity [202]. As we discussed in Section 3.1, polyphenols and MPNs can exhibit both ROS‐scavenging/antioxidant activities or, conversely, ROS production depending on environmental and chemical conditions. These include factors such as pH, the presence and type of coordinated metal ions, oxygen availability, and redox‐active substrates, which can shift the behavior of polyphenols to act as an antioxidant or a pro‐oxidant.

### 3.8 Large‐Scale Implications of Polyphenols in Agricultural Applications

A key challenge to the scale‐up of polyphenol‐based materials in agriculture is the batch‐to‐batch variability of natural polyphenol feedstocks. Commercial TA and technical lignins should be treated as heterogeneous functional feedstocks rather than single‐molecule reagents, as their composition depends on plant species, geographical origin, extraction, and storage conditions [203]. For example, although the chemical structure of TA is commonly represented as decagalloyl glucose (C_76_H_52_O_46_), commercial TA typically consists of a mixture of polygalloyl glucose molecules with different degrees of esterification, and its composition may further change during storage due to aging. Seasonal variation introduces additional variability, as plant polyphenol composition and phenolic hydroxyl density can vary across growing seasons [204]. Such variations may influence coordination and cross‐linking stoichiometry because material formation is governed by the abundance and accessibility of reactive phenolic hydroxyl groups, molecular‐weight distribution, and degree of polymerization, rather than by the total polyphenol mass alone.

To overcome batch‐to‐batch variability, natural polyphenol extracts should be transformed into functionality‐defined feedstocks. Depending on the intended application, polyphenols can be purified to narrow compositional distributions before subsequent chemical modification or materials processing. Homogenization through controlled extraction, blending, and standardized storage conditions may further reduce variability. Furthermore, functional equivalence plays a key role in material design (rather than mass loading equivalence). For example, instead of using metal–polyphenol mass ratio, scalable formulations should be adjusted to the metal ion or cross‐linker content according to batch‐specific functional descriptors, such as phenolic hydroxyl content (catechol and galloyl groups) and metal‐binding capacity.

It is also important to establish standardized characterization methods to enhance reproducibility across production batches and laboratories. Comprehensive characterization of each batch using analytical techniques such as high‐performance liquid chromatography and liquid chromatography–mass spectrometry can be employed to assess composition and purity before further reactions are performed [205, 206]. As the formation of polyphenol‐based materials is also governed by the reactive functional groups, a more robust strategy is to normalize polyphenols based on the abundance of their reactive functional groups (e.g., catechol, galloyl, or pyrogallol moieties) rather than total polyphenol concentration. After phosphitylation of hydroxyl groups, quantitative ^31^P NMR spectroscopy can distinguish and determine the content of different hydroxyl groups in lignin‐ and tannin‐rich feedstocks in a single spectrum [207]. Moreover, the Folin–Ciocalteu assay is a well‐established colorimetric method for the quantitative determination of total polyphenol content based on the reducing capacity of phenolic compounds [208]. Correlating batch‐specific functional descriptors with key material attributes, such as cross‐linking density, coating thickness, release kinetics, and mechanical properties, would be useful to establish structure–property relationships and optimize manufacturing protocols through integration of artificial intelligence or machine learning.

## 4 Environmental and Economic Implications of Polyphenol‐Enabled Materials

For agricultural applications, polyphenol‐enabled materials must be assessed beyond laboratory performance, with attention to environmental behavior, biosafety to mammals and non‐target organisms, and translational feasibility. This section discusses these environmental and techno‐economic factors to clarify how polyphenol‐enabled materials can progress from material design to realistic agricultural applications.

### 4.1 Environmental Fate and Ecological Implications

Conventional petroleum‐derived materials (e.g., synthetic polymers and non‐biodegradable chemicals) used in agricultural applications can cause secondary environmental burdens due to their persistence and accumulation in the environment. Polyphenol‐enabled materials provide a potential alternative due to their inherent biodegradability and environmental compatibility. Polyphenols, as important plant secondary metabolites, contribute to plant defense against biotic and abiotic stresses [209, 210]. For example, polyphenol input can increase soil microbial alpha‐diversity and enzyme activity [211], recruit beneficial microbes, and suppress opportunistic pathogens, thereby reshaping rhizosphere function and plant nutrition [212]. However, their environmental behavior, including their cycling, transformation, and potential ecological impacts in agricultural systems, remains to be evaluated. Polyphenols influence ecosystems across scales, from molecular redox reactions to landscape‐scale biogeochemical feedback. Traditionally viewed as inhibitors of organic matter decomposition, polyphenols are now recognized as active participants in soil carbon cycling, with their roles strongly shaped by microbial metabolism and local redox conditions. Studies in peatland and permafrost systems demonstrate that condensed and monomeric phenolics regulate both oxidative and reductive processes, thereby shaping carbon dioxide and methane fluxes and challenging the classical enzyme‐latch paradigm (Figure 15a) [213]. Furthermore, McGivern et al. have demonstrated that polyphenols can be actively depolymerized and metabolized by soil microorganisms under anoxic conditions, challenging the classical enzyme‐latch paradigm that predicts their accumulation and inhibitory effects. They have also shown that polyphenols are transformed into monomeric and smaller polyphenols, sustaining microbial activity in oxygen‐limited soils. These findings indicate that polyphenols can participate in, rather than suppress, soil carbon cycling under anoxic conditions (Figure 15b) [214]. Similar mechanisms govern nutrient cycling and microbial community structure in soil using polyphenols. Catechins, such as EGCG, selectively inhibit nitrification, while exerting only moderate effects on denitrification, which collectively reduce nitrate accumulation and nitrous oxide emissions [193]. However, prolonged polyphenol accumulation can also cause ecological stress. In aged tea plantations and long‐term monoculture systems, excessive phenolic residues have been associated with soil acidification, shifts in microbial community composition, and reduced ecosystem resilience [215].

**FIGURE 15:**
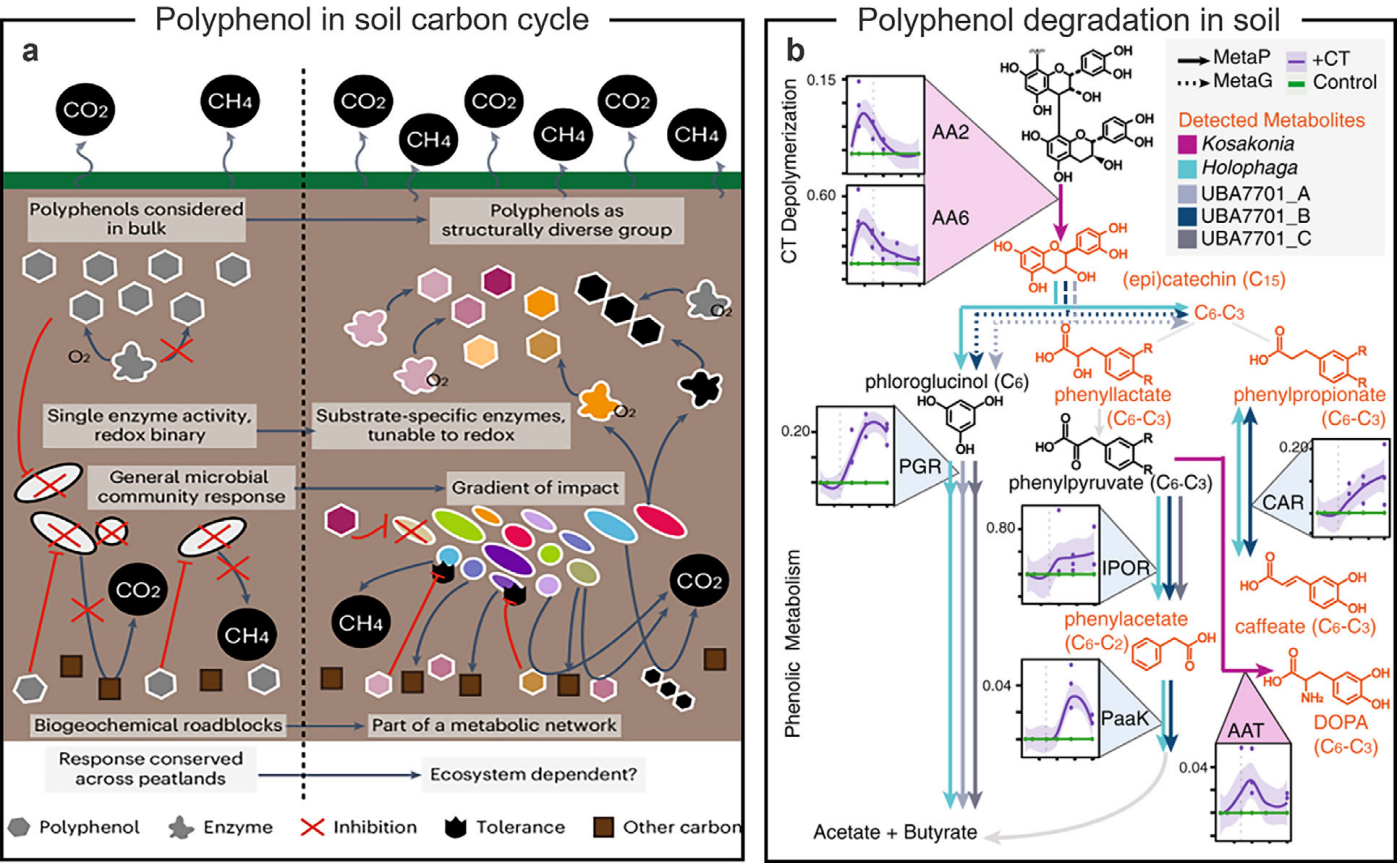
Environmental implications of polyphenols in agriculture. (a) Schematic summarizing the polyphenol lock paradigm, demonstrating the pathways polyphenols could control microbial carbon transformations in anoxic soils. Adapted with permission [213]. Copyright 2024, McGivern et al. (b) Metaproteome data supporting polyphenol degradation by *Kosakonia*, *Holophaga*, and Sporomusales UBA7701. MetaP, metaproteome; MetaG, metagenome; CT, condensed tannin; UBA7701_A/B/C, metagenome‐assembled genome representing an uncultured bacterial lineage; AA2, peroxidase; AA6, 1,4‐benzoquinone; PGR, phloroglucinol reductase; CAR, caffeate–CoA reductase; IPOR, indolepyruvate oxidoreductase; PaaK, penylacetate–CoA ligase; AAT, aromatic amino acid aminotransferase. Adapted with permission [214]. Copyright 2021, McGivern et al.

The introduction of engineered polyphenol‐based materials raises questions regarding their environmental fate, degradation behavior, and potential ecological impacts. In soil, polyphenols undergo dynamic and environment‐dependent processes, including microbial transformation, metal coordination and complexation with proteins, biopolymers, and soil organic matter [210, 214, 216]. For polyphenol–metal coordinated materials, competing soil ligands may further shift the coordination equilibrium. For example, root‐derived carboxylates, such as citrate and malate, can enhance the solubility of Fe, Zn, Mn, and Cu [217, 218, 219]. In addition, humic and fulvic acids can compete for metal ions in a metal‐ and pH‐dependent manner, exhibiting distinct binding affinities and coordination modes for different metal ions (e.g., Cu^2+^ and Pb^2+^), with binding generally increasing with increasing pH [220, 221]. These interactions may promote the disassembly of MPNs, but may also provide opportunities to engineer soil‐responsive materials with controlled stability and release behavior by varying metal species and soil‐specific conditions (e.g., pH, ligand composition). Moreover, polyphenol–metal coordination can improve the robustness of engineered materials during storage and handling. For example, MPN coatings have been shown to preserve microbial viability under high‐temperature and high‐humidity storage stresses, indicating that polyphenol–metal assemblies can withstand harsh environmental stress relevant to agricultural production and application [138]. Therefore, the thermodynamic stability of polyphenol–metal coordinated materials in soil should be considered as a coordination equilibrium governed by multiple environmental factors. Their thermodynamic stability should be quantified using dissociation constants or stability constants under well‐defined soil conditions, including pH, organic matter content, soil texture, and the composition and concentration of competing metal ions and ligands. Furthermore, their kinetic stability should be evaluated by monitoring the rates of metal release, ligand exchange, and coating integrity under representative soil conditions. Meanwhile, biodegradable matrices incorporating polyphenols into polysaccharides or protein matrices exhibit strong antioxidant and antimicrobial activity, while undergoing mineralization under composting or soil conditions, thereby mitigating microplastic accumulation [222]. Furthermore, polyphenols contribute to environmental protection through metal chelation and redox buffering, thereby reducing the bioavailability of toxic ions in contaminated soil [223]. Nevertheless, the long‐term stability, degradation pathways, and ecological effects of polyphenol‐based materials in real soil fluids are poorly understood, particularly in chemically stressed soils such as those contaminated with heavy metals or affected by salinization. Moreover, the available database of dissociation (or stability) constants for metal ion–polyphenol complexes, including polyphenols with different p*K*_a_ values, should be evaluated prior to experimental design. This would facilitate the rational selection of polyphenols for targeting specific metal ions under varying soil conditions, as the optimal polyphenol–metal ion combinations may vary, depending on the various soil environments, such as more stable Fe^3+^–TA formulation for organic‐matter‐rich soils with strong ligand competition.

Beyond their fate within soil, polyphenol‐enabled materials and their degradation products may enter humans and animals through food‐chain transfer via dietary intake, as well as through occupational exposure via dermal contact or inhalation during handling and application [224, 225, 226]. Dedicated in vivo studies of polyphenol materials investigating pharmacokinetic, biodistribution, or toxicological properties following these specific routes remain limited. The evidence below instead draws on the routes in which polyphenol‐based systems have been most extensively characterized. Although these conditions do not closely resemble agricultural exposure, these studies offer some guidance on their potential biosafety.

Tea polyphenols and related plant‐derived phenolic ligands (e.g., quercetin, resveratrol, and curcumin) are well known for their antioxidant, antitumor, anti‐inflammatory, and lipid‐lowering activities [227, 228, 229]. Studies indicate that the biological effects of TA are markedly concentration‐dependent, shifting from antioxidant and anti‐inflammatory at low doses to cytotoxic at higher concentrations [230]. Nevertheless, across the more rigorously controlled and dose‐defined studies surveyed below, polyphenol‐based nanoformulations have generally shown favorable safety profiles at doses examined. For example, EGCG‐ and curcumin‐capped nanoparticles have been characterized using a physiologically based pharmacokinetic model built on mouse organ‐content data collected out to 56 days, providing quantitative clearance and tissue‐partitioning parameters alongside interspecies extrapolation [231]. Systemic toxicity has likewise been examined across several injury models: EGCG‐containing nanoparticles attenuated cytokine and histopathological markers in a diquat‐induced kidney injury model [232], quercetin‐containing nanoparticles improved renal biochemistry and histology in an ischemia/reperfusion acute kidney injury model [233], and resveratrol‐containing nanocapsules reduced pulmonary inflammation in a lipopolysaccharide‐induced lung injury model [234], with no carrier‐related adverse effects observed at the doses examined. Furthermore, long‐term studies, spanning 90 days to 1 year in rodents [235], and humans [236], together with a formal regulatory safety opinion from the European Food Safety Authority [237], provide additional evidence for the absence of cumulative organ toxicity at doses exceeding typical dietary intake.

Among polyphenol‐based platforms, metal coordination is one of the most widely used strategies to construct MPNs such as coatings, capsules, or nanoparticles. The incorporation of metal ions introduces additional safety considerations for MPN platforms. Pharmacokinetic characterization has shown that Fe^3+^–shikonin MPN nanomedicines prolong antigen blood circulation half‐life roughly sixfold relative to the free antigen following intravenous administration in mice, without causing observable organ damage or abnormal liver and kidney function indices [238]. Biodistribution has been directly quantified by inductively coupled plasma‐mass spectrometry or mass spectrometry in several MPN platforms—Zr tracking after intravenous administration of mRNA‐MPN nanoparticles in mice [239] and Ti tracking in major organs following in‐situ gelation of a Ti^4+^–TA hydrogel [240]. Systemic toxicity has been assessed via bronchoalveolar cytokines, hematology, liver‐enzyme levels, and lung histology following nebulized delivery of Fe^3+^–TA capsules [241], and via biocompatibility testing across cells, blood, zebrafish, and mice for a topically applied antimicrobial MPN nanoparticle [120], with no significant toxicity reported in either study. Medium‐ and long‐term accumulation data remain limited. Repeated intraperitoneal dosing of Fe^3+^–TA nanoparticles over 28 days to 10 weeks has shown no evidence of genotoxicity on liver micronucleus assay or of early‐phase hepatocarcinogenicity [242]; a 30‐day extension of the nebulized Fe^3+^–TA capsule study has similarly revealed no cumulative toxicity [241]. Likewise, a 14‐week in‐situ gelation study of a Ti^4+^–TA hydrogel has demonstrated no adverse distal‐tissue accumulation despite detectable titanium biodistribution [240]. Within an agricultural context, Fe^3+^–TA pesticide nanocapsules have shown lower cytotoxicity toward human liver cells and reduced acute toxicity toward bees relative to technical pesticide formulations [147]. Cu^2+^‐enabled MPN nanopesticides have similarly shown lower acute toxicity in zebrafish than a commercial Cu‐based fungicide and with no significant adverse effects on lettuce growth, nutrient composition, microelement uptake, or photosynthetic parameters in hydroponic cultivation [145]. Together, these findings indicate that rationally designed MPN platforms can exhibit favorable biosafety profiles, while reducing risks to non‐target organisms and crops under field‐relevant conditions.

Collectively, these findings indicate that polyphenol‐based platforms, which are predominantly studied in assemblies rather than as free polyphenols, generally display favorable short‐to‐medium‐term biocompatibility. However, the long‐term consequences of ingestion‐based and environmentally mediated exposure remain largely unknown. In particular, the fate, biotransformation, and potential toxicity of degradation products released as these materials decompose under field conditions, represent essential knowledge gaps that need to be addressed before agricultural translation.

### 4.2 Techno‐Economic Considerations of Polyphenol‐Enabled Materials

Techno‐economic analysis is essential for assessing the translational potential of polyphenol‐enabled materials for agricultural applications, where new products are typically benchmarked against low‐cost raw materials and mature industrial formulations [243, 244]. Unlike conventional petroleum‐derived polymers, the overall cost of polyphenol‐based materials is determined not only by the final material price, but also by feedstock selection, extraction, purification, chemical modification, solvent use, drying, and formulation processing [245, 246]. Therefore, translating polyphenol‐enabled agricultural materials from laboratory scale to field deployment should prioritize low‐cost or minimally purified feedstocks, high‐yield and solvent‐efficient extraction methods, simplified synthesis, and manufacturing routes compatible with existing industrial processes such as coating, granulation, and spray drying.

From a materials‐design perspective, cost reduction should not rely on a single strategy, but should be achieved through application‐specific optimization, as the required material input and the functional performance vary across different agricultural systems. For high‐volume applications, such as CRF coatings, soil conditioners, and contaminant immobilization, material costs are amplified at field scale. Low‐cost and scalable feedstocks, such as TA, lignin‐rich industrial by‐products, and crude plant extracts, are highly suitable for these agricultural application scenarios [247]. These materials may provide an appropriate balance between functionality and economic viability. Lignin is attractive due to its abundance as an aromatic by‐product from pulping and biorefinery processes, whereas TA offers high aqueous solubility and chemical reactivity for reproducible coating and coordination assembly. In addition to feedstock selection, simplifying material synthesis through one‐pot reactions, self‐assembly, or dynamic supramolecular interactions may further reduce reagent consumption, processing complexity, and energy input. For medium‐volume applications, such as seed coatings, foliar coatings, and food preservation, feedstock selection should balance cost with adhesion, barrier performance, redox activity, and biological compatibility. In contrast, low‐volume, high‐functionality applications, including pesticide delivery and biosensing, may justify the use of more defined or modified polyphenols when precise interfacial chemistry, cargo loading, colloidal stability, or environmental responsiveness is essential for functional performance. In these systems, maximizing functionality per unit mass through hierarchical/composite structures may reduce overall material consumption and partially offset higher production cost.

Polyphenol‐enabled materials may not outperform traditional petroleum‐derived polymers solely on manufacturing cost. Their competitive techno‐economic value should instead be evaluated according to whether their added functions generate measurable agronomic and environmental benefits. For example, Fe^3+^–TA‐modified polymer‐coated urea increased rice yield by 8.3%, reduced reactive nitrogen losses by nearly 24%, and improved net ecosystem economic benefits by nearly 11% compared with conventional urea at the same nitrogen rate [175]. These findings demonstrate that the economic value of polyphenol‐enabled materials arises from improved nutrient use efficiency, reduced input losses, and enhanced crop productivity rather than lower material cost alone. Therefore, future evaluations should integrate techno‐economic assessments with life cycle assessment and function‐normalized metrics (such as application frequency, crop yield, greenhouse‐gas mitigation, and environmental externalities) to identify application scenarios where multifunctionality and sustainability confer measurable economic benefits. These would help facilitate the translation of polyphenol‐enabled materials to commercially viable agricultural technologies.

## 5 Challenges and Future Outlook for Agriculture

The application of polyphenol‐enabled materials in agriculture has shown significant promise across a range of functions, including crop protection, nutrient delivery, soil remediation, food preservation, sensing, and seed coating. In this section, we outline the key challenges in agriculture and give an outlook covering precision and data‐driven agriculture, environmental and ecosystem protection, climate‐resilient plant interfaces, and circular bioeconomy and sustainable manufacturing, providing a framework for next‐generation agricultural materials.

### 5.1 Key Challenges in Agriculture

More than 40% of the world's arable land is used for agriculture, and the low precision and high labor intensity associated with large‐scale farming are the primary constraints on current agricultural transformation [1]. The application and activation of agrochemicals are largely uncontrolled, leading to nonspecific distribution, low targeting efficiency, and substantial losses through leaching, volatilization, and off‐target deposition [2]. Therefore, a significant fraction of inputs (e.g., pesticides, fertilizers) fails to reach the intended plant–soil interface, causing both reduced utilization efficiency and environmental contamination. To compensate for this inefficiency, repeated applications and manual interventions are required to maintain crop production, resulting in labor‐intensive practices and increased economic burdens. Fundamentally, this limitation arises from the inability of conventional agrochemicals to adapt to the spatial heterogeneity and temporal variability of soil and climatic conditions, preventing precise regulation of when and where active compounds are released and activated. Addressing these challenges requires the development of precision and smart agricultural systems in which material platforms can dynamically respond to environmental and biological stimuli, thereby enabling controlled delivery and on‐demand release of agrochemicals at the plant–soil interface (Figure 16).

**FIGURE 16:**
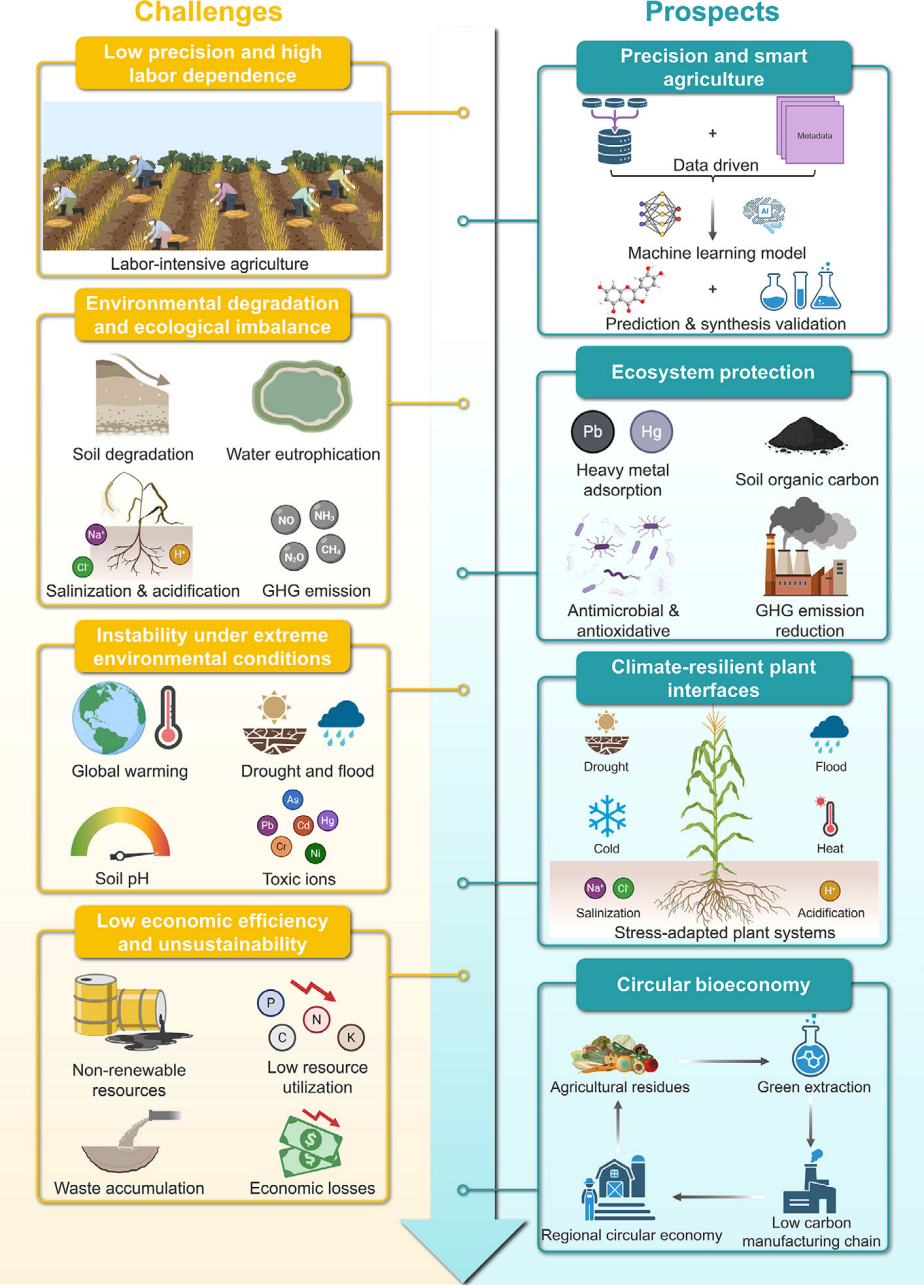
Key challenges in agriculture and prospects for polyphenols in agriculture. GHG, greenhouse gas. Created in BioRender.com. Liu, H. (2026) https://BioRender.com/dl6fntx.

Most agrochemicals are lost from the plant–soil system through leaching, runoff, and volatilization, leading to soil degradation, water eutrophication, and increased greenhouse gas emissions. The accumulation of persistent polymers and toxic pesticides disrupts physicochemical stability, causing salinization, acidification, and loss of organic matter and fertility. Nutrient leaching and runoff drive eutrophication in aquatic systems, while microbial transformation of excess nitrogen accelerates emissions of greenhouse gases such as N_2_O [248]. These processes destabilize agroecosystem function and reduce long‐term sustainability. These challenges originate from the lack of control over the retention, transformation, and environmental fate of agricultural inputs. The widespread use of nonbiodegradable and petroleum‐derived materials further introduces persistent residues, exacerbating ecological imbalance (Figure 16).

Agricultural systems lack stability under extreme environmental conditions. Variations in temperature, water availability, soil pH, and ionic composition alter the stability, reactivity, and delivery behavior of agrochemicals, leading to premature degradation, uncontrolled release, and loss of efficacy [249]. These effects are amplified under global warming, drought, flooding, and soil physicochemical fluctuations, resulting in inconsistent performance across field conditions. This limitation originates from the inability of conventional agrochemicals to maintain functionality while adapting to environmental variability. Without mechanisms to buffer or respond to external stresses, agrochemical performance remains highly dependent on fluctuating conditions, hindering the development of resilient agricultural systems (Figure 16).

Agricultural systems exhibit low economic efficiency and poor sustainability due to inefficient and noncircular resource use. A large proportion of agricultural inputs is derived from nonrenewable sources with low utilization efficiency, substantial losses during application, and environmental dissipation. These inefficiencies lead to the accumulation of residues in soil and water, generating waste streams and secondary environmental burdens. Moreover, repeated application and resource loss increase operational costs, reducing economic efficiency. This challenge originates from the mismatch between resource input, utilization, and recovery within current agricultural systems. The absence of closed‐loop strategies for resource retention, recovery, and reuse limits long‐term sustainability and economic viability (Figure 16).

### 5.2 Future Prospects for Polyphenols in Agriculture

Future progress in polyphenol‐based agricultural materials will depend on bridging molecular innovation with system‐level sustainability and data‐driven management. As research transitions from laboratory studies to real agricultural systems, four primary future directions are expected to define the next stages of development (Figure 16).

#### 5.2.1 Precision and Data‐Driven Agriculture

Precision and data‐driven agriculture will become a key frontier [250]. Polyphenol‐enabled smart delivery systems can couple signal‐responsive release with real‐time soil monitoring to achieve adaptive nutrient and pesticide management. Quantitative release modelling will be essential for translating cumulative release profiles into mechanistic and design‐relevant parameters. Linking kinetic parameters with material properties can guide the design of precision‐release agricultural material platforms tailored to different environmental conditions. The inherent chemical versatility of polyphenols, including dynamic coordination, reversible redox activity, and stimuli‐responsive bonding, enables materials to respond sensitively to chemical stimuli (e.g., pH and redox potential). By integrating these responsive mechanisms with digital farming platforms, polyphenol‐enabled materials can modulate the timing, location, and rate of agrochemical release in response to environmental signals, thereby enabling tunable‐rate applications that better align input with plant demand. Specifically, metal coordination can act as reversible gates for controlled release, while redox‐active phenolic groups can buffer oxidative environments and regulate release behavior under stress conditions. The convergence of polyphenol chemistry, sensing technologies, and computational analytics is anticipated to transform agrochemical use from static formulations into adaptive and self‐regulating systems, enabling more precise, efficient, and sustainable agricultural management.

#### 5.2.2 Environmental and Ecosystem Protection

Environmental and ecosystem protection represents another central opportunity [251]. The multifunctional reactivity of polyphenols enables direct regulation of key environmental processes. Through strong metal coordination, polyphenol‐based materials can immobilize toxic metal ions and reduce their bioavailability. Their redox‐active phenolic structures can modulate soil redox conditions and scavenge reactive species, contributing to the mitigation of greenhouse gas emissions and the stabilization of soil organic carbon. Embedding these functions within soil amendments, coatings, and remediation matrices enhance nutrient retention and suppress contaminant mobility at the soil interface. In addition, the antimicrobial and antioxidative properties of polyphenols can regulate microbial transformations involved in nutrient cycling and alleviate oxidative stress, supporting soil ecological stability. These coupled chemical and biological effects position polyphenol‐based materials as promising platforms for ecosystem restoration and low‐emission agricultural practices. However, realizing these environmental benefits will require parallel attention to the biosafety of the materials themselves. In particular, the environmental fate of degradation products, as well as their long‐term biodistribution, systemic toxicity, and accumulation in non‐target organisms, humans, and the broader food chain, remain insufficiently characterized under agricultural exposure conditions. Addressing these knowledge gaps will require assessing platforms that mimic realistic field conditions, such as soil‐column or lysimeter‐based mesocosm systems with native microbial communities, temperature cycling, and UV irradiation. Coupling these with isotope‐ or fluorophore‐labeled tracers will allow tracking of degradation products and released metal ions across soil, leachate, and plant tissue. These approaches would generate the exposure and mass‐balance data currently lacking in biosafety assessments, providing a basis for predictive environmental fate models analogous to those already established for conventional agrochemicals.

#### 5.2.3 Climate‐Resilient Plant Interfaces

Climate‐resilient plant interfaces provide a promising pathway to protect crops from abiotic stresses linked to global climate change. Polyphenol‐derived seed coatings, foliar films, and root‐zone barriers can provide antioxidative protection, regulate water exchange, and maintain adhesion under thermal or osmotic stress. Moreover, the redox‐active phenolic groups can scavenge oxidative species (e.g., H_2_O_2_) and buffer stress‐induced redox imbalances, while hydrogen‐bonded networks and hydrophobic domains regulate water retention and transport under drought or salinity conditions. Such dynamic and stimuli‐responsive networks allow polyphenol‐derived seed coatings, foliar films, and root‐zone barriers to adapt to environmental changes, maintaining functional performance under fluctuating conditions. Future efforts should integrate molecular‐level design with plant physiological responses to develop adaptive and resilient agricultural interfaces.

#### 5.2.4 Circular Bioeconomy and Sustainable Manufacturing

Circular bioeconomy and sustainable manufacturing will underpin long‐term scalability and adoption. Agricultural and forestry residues (e.g., lignin, tannins, and crop waste) provide renewable polyphenol feedstocks that can be converted into functional materials through mild processes such as extraction and self‐assembly. These materials can be engineered into biodegradable coatings and controlled‐release systems that reintegrate into soil after use. For example, polyphenol‐enabled coatings can degrade into phenolic species that participate in soil carbon cycling, reducing residue accumulation. Local processing and direct application of polyphenols enable the conversion of agricultural residues into functional materials with reduced processing and waste.

Cultivating polyphenol‐rich crops in space is emerging as a realistic strategy to deliver not only calories but also functional metabolites that support human health and system sustainability under extreme environments through a circular bioeconomy. Space‐based agriculture will rely on controlled environment platforms (hydroponics/aeroponics) where polyphenol biosynthesis can be precisely regulated through metabolic programming using spectral engineering (blue/UV‐A light), CO2 control, and mild elicitation protocols (e.g., short pre‐harvest nutrient or osmotic stress pulses). This approach enables a two‐stage operation in which biomass is first maximized under optimal growth conditions, followed by targeted induction of the phenylpropanoid pathway to enrich flavonoids, tannins, and anthocyanins without excessive yield penalties. Moreover, translation requires addressing microgravity‐specific constraints, including altered root‐zone aeration and water distribution, higher sensitivity to microbial contamination, and potential shifts in redox metabolism under radiation exposure. Looking forward, polyphenol‐rich crops could serve as dual‐use biological resources in closed‐loop habitats, providing antioxidant‐rich food while also supplying plant‐derived polyphenols for in situ production of bioactive coatings, antimicrobial surfaces, and nutrient‐stabilizing matrices. Such integration positions polyphenols as a bridge between space nutrition and sustainable biomaterials engineering.

In summary, significant progress has been made in advancing the molecular design, assembly strategies, and multifunctional performance of polyphenol‐inspired materials for agricultural applications. We anticipate that these polyphenol‐based materials will play a pivotal role in shaping next‐generation materials for agriculture from fabricating advanced devices in smart agricultural technologies to protecting ecosystems and ensuring global food security.

## Conflicts of Interest

The authors declare no conflicts of interest.

## Data Availability

Data sharing not applicable to this article as no datasets were generated or analysed during the current study.
